# Content and Quality of Infant Feeding Smartphone Apps: Five-Year Update on a Systematic Search and Evaluation

**DOI:** 10.2196/17300

**Published:** 2020-05-27

**Authors:** Heilok Cheng, Alison Tutt, Catherine Llewellyn, Donna Size, Jennifer Jones, Sarah Taki, Chris Rossiter, Elizabeth Denney-Wilson

**Affiliations:** 1 Susan Wakil School of Nursing and Midwifery Faculty of Medicine and Health The University of Sydney Camperdown Australia; 2 Centre of Research Excellence in Early Prevention of Obesity in Childhood The University of Sydney Camperdown Australia; 3 Child and Family Health Nursing Sydney Local Health District NSW Health Camperdown Australia; 4 Lactation Clinic Royal Prince Alfred Hospital NSW Health Camperdown Australia; 5 Sydney Institute for Women, Children and their Families Sydney Local Health District NSW Health Camperdown Australia; 6 Health Promotion, Population Health Research & Evaluation Hub Sydney Local Health District NSW Health Camperdown Australia; 7 Sydney School of Public Health Faculty of Medicine and Health The University of Sydney Camperdown Australia

**Keywords:** breast feeding, bottle feeding, infant food, readability, consumer health information, breastfeeding, mobile apps, smartphones

## Abstract

**Background:**

Parents use apps to access information on child health, but there are no standards for providing evidence-based advice, support, and information. Well-developed apps that promote appropriate infant feeding and play can support healthy growth and development. A 2015 systematic assessment of smartphone apps in Australia about infant feeding and play found that most apps had minimal information, with poor readability and app quality.

**Objective:**

This study aimed to systematically evaluate the information and quality of smartphone apps providing information on breastfeeding, formula feeding, introducing solids, or infant play for consumers.

**Methods:**

The Google Play store and Apple App Store were searched for free and paid Android and iPhone Operating System (iOS) apps using keywords for infant feeding, breastfeeding, formula feeding, and tummy time. The apps were evaluated between September 2018 and January 2019 for information content based on Australian guidelines, app quality using the 5-point Mobile App Rating Scale, readability, and suitability of health information.

**Results:**

A total of 2196 unique apps were found and screened. Overall, 47 apps were evaluated, totaling 59 evaluations for apps across both the Android and iOS platforms. In all, 11 apps had affiliations to universities and health services as app developers, writers, or editors. Furthermore, 33 apps were commercially developed. The information contained within the apps was poor: 64% (38/59) of the evaluations found no or low coverage of information found in the Australian guidelines on infant feeding and activity, and 53% (31/59) of the evaluations found incomplete or incorrect information with regard to the depth of information provided. Subjective app assessment by health care practitioners on whether they would use, purchase, or recommend the app ranged from poor to acceptable (median 2.50). Objective assessment of the apps’ engagement, functionality, aesthetics, and information was scored as acceptable (median 3.63). The median readability score for the apps was at the American Grade 8 reading level. The suitability of health information was rated superior or adequate for content, reading demand, layout, and interaction with the readers.

**Conclusions:**

The quality of smartphone apps on infant feeding and activity was moderate based on the objective measurements of engagement, functionality, aesthetics, and information from a reliable source. The overall quality of information on infant feeding and activity was poor, indicated by low coverage of topics and incomplete or partially complete information. The key areas for improvement involved providing evidence-based information consistent with the Australian National Health and Medical Research Council’s Infant Feeding Guidelines. Apps supported and developed by health care professionals with adequate health service funding can ensure that parents are provided with credible and reliable resources.

## Introduction

### Background

Parents of a new baby can access a wealth of information and support from the internet through multiple electronic devices [1-3]. There is evidence to suggest, however, that web-based advice is not always evidence-based, even in the critical area of infant nutrition [4]. Smartphone ownership has expanded worldwide, with 81% of Australian adults and 97% of Australians aged 18 to 34 years owning a smartphone in 2018 [5]. Recent national data suggest that 46% of Australian adults accessed the internet for health information [6]. In 2017, 84% of Australian adults used mobile phones to access the internet, exceeding access through laptop computers (69%) and desktop computers (54%) [7]. Furthermore, smartphone ownership has now surpassed the ownership of desktop and laptop computers [8].

The proliferation of web-based health information sources is reflected by the growing literature for health care professionals discussing and advising the use of new technology [9-12]. Studies have shown that parents and pregnant women trusted hospital, government, and university websites as accurate, regulated, useful, and current sources of pregnancy and parenting information [13,14]. Parents in a video education study on introducing solid foods preferred the internet as a source of information for infant nutrition and felt that public authorities were important information providers [15].

Governments and nonprofit organizations have developed smartphone apps to promote and enhance breastfeeding [16-20]. A recent content analysis of social support in 31 breastfeeding apps found that the most common topics were managing breastfeeding problems (informational support) and locating where to express or breastfeed in public (instrumental support) [21]. Increasingly, clinical trials have assessed mobile health (mHealth) and smartphone apps as interventions to promote and support breastfeeding among mothers and their partners [22-24] through text messaging, goal setting, access to information and videos, provision of online support groups, and troubleshooting breastfeeding difficulties. A review by Tang et al [25] on digital interventions that support breastfeeding found that client communication systems to communicate breastfeeding information, facilitate communication, and provide on-demand information services through text messages, phone calls, email, smartphone apps, and websites may improve breastfeeding adherence.

In Australia, government health services use mHealth apps to support routine child and family health nursing practice in fields such as child literacy and development [26,27], immunization [28], and safe infant sleeping [29]. An early childhood obesity prevention trial will test an app developed for parents and caregivers [30] to be integrated into an Australian statewide pregnancy coaching service [31]. Clearly, apps used as part of routine care must meet practice standards for providing understandable, reliable, current, and evidence-based information independent of commercial associations. This highlights the need for evidence-based, well-developed, and updated apps.

Despite the proliferation of apps and their increasing popularity with parents, their quality may not have kept pace with their quantity. Earlier research on infant feeding apps and websites available in Australia found that 78% of the apps were of poor quality, with deficits in the breadth and completeness of information, author credibility, and readability [4]. A similar review of infant feeding apps in China found that most apps advertised infant formula and parenting products and rated poorly on the availability of information, author credibility, and transparency in disclosing advertising policy, app ownership, and app sponsorship [32]. A recent review of mHealth apps for parents of infants in neonatal intensive care units found that smartphone apps were functional but had low quality and credibility, with only 2 apps rated *good* by nurse and information scientist reviewers and only 5 apps deemed *trustworthy* [33].

Since the 2015 study [4], the Australian ownership of smartphones has increased from 64% to 81%, and the proportion of users accessing the internet through mobile phones has increased from 42% to 84% [7,34]. In 2017, there were 325,000 health, fitness, and medical apps available [35]. The increasing interest in infant feeding and physical activity apps indicated by popular search queries in the Google search engine (Multimedia Appendix 1) demonstrates the need to systematically assess and update the current smartphone app landscape. Furthermore, there is a continual turnover of smartphone apps, with several apps from the 2015 study [4] subsequently removed from distribution.

### Aim

Given the rapid expansion of digital technology into the realm of child health, this study aimed to evaluate the quality of information on infant nutrition and physical activity currently available to Australian parents via smartphone apps. It updates and expands the 2015 systematic assessment, examining the comprehensibility, suitability, and readability of information in free and paid apps available in Australia.

## Methods

### Study Design

This study used systematic methods to identify, select, and evaluate infant feeding and activity apps that were available in Australia between August 2018 and January 2019. It assessed aspects of their quality and utility using validated and purpose-specific instruments to replicate and update an earlier study [4]. Full details of the methods are given in Multimedia Appendix 2, and evaluation tools are described in Multimedia Appendix 3.

#### Stage 1: App Selection

Smartphone apps were identified by searching app platforms from the two largest smartphone operating systems: App Store for iPhone Operating System (iOS; Apple Inc) and Google Play for Android (Google LLC). The search terms included variations in *infant feeding, baby feeding, breastfeeding, formula feeding, bottle feeding, baby food, baby weaning, infant activity,* and *tummy time.*

We used the search engine Google Play on a desktop for searching Android apps [36]. It was not possible to search the App Store on a desktop [37]; therefore, we conducted all App Store searches on the authors’ iOS smartphones.

Members of the research team screened all located apps for eligibility: 4 authors screened iOS apps, and 2 authors screened Android apps. The first author cross-checked all apps. Apps were reviewed if they met the inclusion criteria. Any disagreements regarding the inclusion of apps in the study were discussed until consensus was reached.

The inclusion criteria for selection included apps written in English, targeted at parents of infants up to one year of age, and containing information on at least one of the following topics: milk feeding behaviors (breastfeeding, formula feeding, expressing breast milk, frequency or timing of feeding, and correct preparation of infant formula), solid food feeding behaviors (age of introduction, types of food introduced, repeated exposure, varied exposure, and reducing exposure to unhealthy food and beverages), or infant activity (tummy time, infant play, and movement).

In the selection stage, we excluded apps that were inaccessible with dead or broken links; were formatted as electronic books, news, magazines, podcasts, blogs, or word documents; had restricted access; did not have an English language option or were machine-translated into English; were games or gaming apps, or contained stolen or farmed content [38] from other apps or websites. Examples of excluded apps are given in Multimedia Appendix 2. Apps whose main function was to monitor or time infant care tasks, without providing any educational information on infant feeding and activity, were also excluded.

#### Stage 2: App Evaluation

In this stage, reviewers evaluated the selected apps on several dimensions (coverage and depth of information, quality, data security, and app accessibility, suitability, and readability) using a range of instruments. The reviewers rated all instruments using the Research Electronic Data Capture platform (Vanderbilt University) [39].

##### Coverage and Depth of Information

Accurate coverage (referred to hereafter as *coverage*) and depth of information were evaluated using a quantitative tool developed for the 2015 study [4], based on the Australian government’s guidelines on infant feeding [40] and physical activity [41], with permission from the tool’s authors. It has 9 topics with 22 subtopics on encouraging and supporting breastfeeding, initiating breastfeeding, establishing and maintaining breastfeeding, managing common breastfeeding problems, expressing and storing breast milk, breastfeeding in specific situations, preparing and using infant formula, introducing solid foods, and encouraging infant activity.

Coverage, defined as the breadth of the subtopics correctly covered in each app, was scored as either correct (+1), incorrect (−1), not addressed (0), or not applicable. Depth, defined as the completeness of information covered in each subtopic, was scored as partially complete (+0.5), complete (+1), or incompletely addressed or incorrect information (0). If the subtopic was scored incorrectly for coverage, it was automatically scored as incorrect for depth.

We also rated each app’s coverage using the criteria from the Health-Related Website Evaluation Form [42]. Coverage was summarized as excellent (≥90%), adequate (75%-89%), or poor (≤74%). Depth was summarized as complete (100%), partial (50%-99%), or low or no (≤49%) completeness.

##### App Quality

App quality was evaluated using the Mobile App Rating Scale (MARS) [43], which was not available at the time of the original study in 2015. The MARS is a 23-item quality rating tool that uses a 5-point rating scale, scored as 1 (inadequate), 2 (poor), 3 (acceptable), 4 (good), and 5 (excellent), with 4 objective scales on engagement (5 domains on interesting, fun, or interactive content), functionality (4 domains on app navigation and logical usability), aesthetics (3 domains on graphic design and visual appeal), and information quality (7 domains on credibility of source). The MARS also includes 1 subjective quality scale incorporating the user’s judgment on their likelihood of recommending, using, and purchasing the app and a personal 5-star rating. This is reported separately as the *subjective MARS score*.

A final measurement of app quality, the objective MARS score, is calculated as a 5-star rating using the mean from the scores from the objective (engagement, functionality, aesthetics, and information quality) scales.

##### App Usability

The authors of the previous study [4] developed 2 additional scales not included in other app assessment tools available or found by the authors during the app evaluation stage. These were data security, with items assessing data encryption and privacy, and accessibility, incorporating multilanguage options, one-handed functionality, and availability of help guides. We added these scales to the MARS tool to create the *modified MARS score*, which we calculated separately from the objective MARS score.

##### Suitability of Information

The appropriateness of the information on the apps was evaluated using the Suitability Assessment of Materials (SAM) tool [44]. The SAM is a 22-item validated instrument that assesses content, literacy level, graphics, layout, interaction with readers, and cultural appropriateness. Each item is scored as superior (+2), adequate (+1), not suitable (0), or not applicable. The sum of the scores of the items generates a final score summarized as superior (70%-100%), adequate (40%-69%), or not suitable (0%-39%) appropriateness of information for the target audience.

The hypothetical target audience used in the app evaluation was Australians with a year 3 to 4 reading level, with or without a multicultural background [45].

##### Readability

The SAM also assesses the readability, or grade level of written text, measured using the Flesch-Kincaid (F-K) [46] and the Simple Measure of Gobbledygook (SMOG) [47] tools. The reviewers assessed the readability by typing a section of writing from each app into an online readability calculator [48] that calculated F-K and SMOG scores; they also used Microsoft Word software (2010 and later; Microsoft Corporation) [49] to generate an alternative F-K score. Each reviewer selected the passage of the text they assessed.

The F-K and SMOG scores are reported as American reading grades. The Australian federal government’s Plain English guidelines [45] recommend writing for a reading level of Australian school year 3 to 4, the equivalent of American Grade 3 to 4 reading level. The South Australian state government’s health literacy guidelines recommend writing for a reading level of Australian year 8 [50].

Readability is an item in the SAM. Using the SAM, we summarized F-K and SMOG readability scores as superior (Grade 5 and lower reading level), adequate (Grade 6 to 8 reading level), or not suitable (Grade 9 or higher reading level).

### Interrater Reliability

We undertook interrater reliability (IRR) testing with 2 or more reviewers assessing at least 20% of the selected apps using all rating tools. We tested apps that were available on both iOS and Android platforms. Discrepancies were discussed until reviewers reached a consensus on their final ratings.

IRR was calculated for the readability scores, MARS scores, SAM, and the evaluation of information content using Krippendorff α (α), which is appropriate when there are missing or incomplete data. Using Krippendorff standards for data reliability, .667≤α<.80 was accepted as tentatively reliable, and α≥.80 was accepted as reliable.

### Statistics

Statistical analyses were performed using IBM SPSS Statistics for Windows, version 25.0 (IBM Corporation). Descriptive results on the ratings from the various instruments are reported as median (IQR). We calculated the correlations among different measures of readability using the Spearman rank-order correlation. We also compared the 5-point rating scores in the MARS tool with the user ratings of the apps presented in the Apple App Store and Google Play Store using the Pearson correlation coefficient. Krippendorff α was calculated using the KALPHA macro for IBM SPSS [51]. Significant values were indicated at *P*<.05.

### Ethics Approval

This study did not require ethics approval.

## Results

### Stage 1: Smartphone App Selection

#### Screening Process

**Figure 1:**
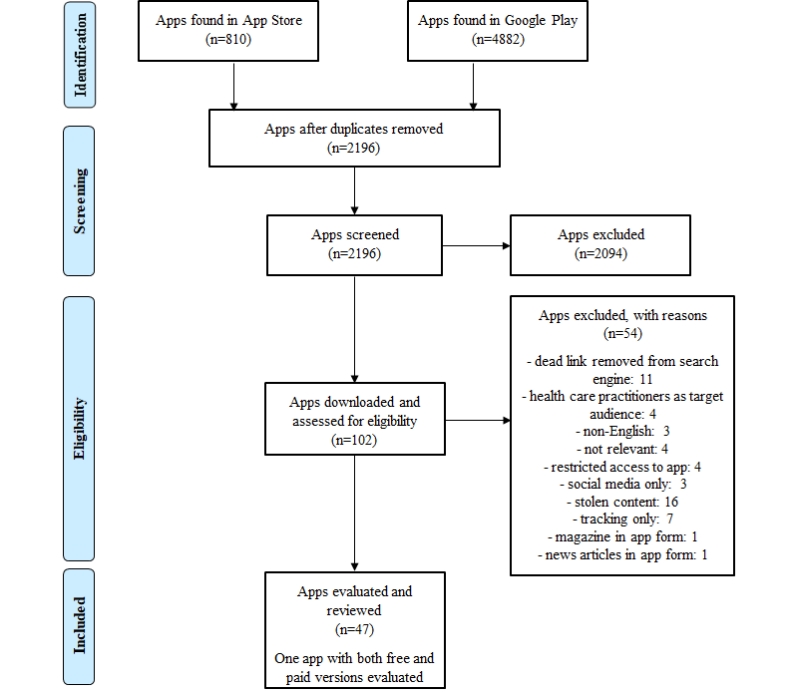
Preferred Reporting Items for Systematic Reviews and Meta-Analyses diagram of smartphone app selection process.

App searches were performed between August and September 2018. A total of 5692 apps were identified for screening (Figure 1), with 3496 duplicates removed and 2196 apps screened for potential inclusion. After screening, 102 apps were downloaded for evaluation. Of these, 54 were excluded, and 47 were reviewed between September 2018 and January 2019. The apps included in this study are described in Multimedia Appendix 4. We undertook 59 evaluations for 47 apps, including 27 and 32 evaluations on iOS and Android smartphones, respectively.

#### Description of the Selected Apps

Overall, 1 app was developed with nationally competitive research funding, 10 apps were developed by the government or had government affiliations, 3 apps were developed by universities or had university affiliations, and 33 were commercial.

Furthermore, 2 apps were trialed with surveys [52,53]. One app was used in a randomized controlled trial but was not objectively evaluated [54]. Of the 47 apps, 35 apps (74%) were free to access, although 12 of these required payment to remove advertisements; access additional content, functions, and information; or access the full app without preview restrictions. Overall, 12 of the 47 apps (26%) were only accessible by purchase. A total of 10 apps were, as reported by app developers, available in languages other than English (eg, Arabic, Bosnian, Chinese, Danish, Dutch, French, German, Hindi, Italian, Japanese, Polish, Portuguese, Russian, Spanish, Swedish, and Vietnamese).

Of the 47 apps, 32 apps (68%) were located through infant feeding–related search terms, 13 (28%) through terms related to introduction to solids, and 25 (53%) through infant activity–related terms. Several apps were found through multiple keyword search terms, for example, in search terms related to infant feeding and introduction to solids.

### Stage 2: Smartphone App Evaluation

#### Interrater Reliability

Overall, 12 of the 47 apps (26%) were evaluated independently by 2 different reviewers using iOS and Android platforms (Multimedia Appendix 4) to assess the IRR.

There was reliable IRR agreement (α≥.80) for the objective MARS ratings and the depth of information content in the infant activity subtopic. There was acceptable IRR agreement (.667≤α<.80) for the modified MARS ratings, depth of information content in the infant feeding subtopic, and coverage of information content in the infant activity subtopic.

Although the IRR scores were relatively low on several instruments, the reviewers discussed discrepancies and the interpretation of evaluation criteria to ensure greater unanimity in future scoring. The following tables present results for 59 evaluations, taking into account the 12 apps rated by the 2 reviewers.

#### Coverage and Depth of Information

**Figure 2:**
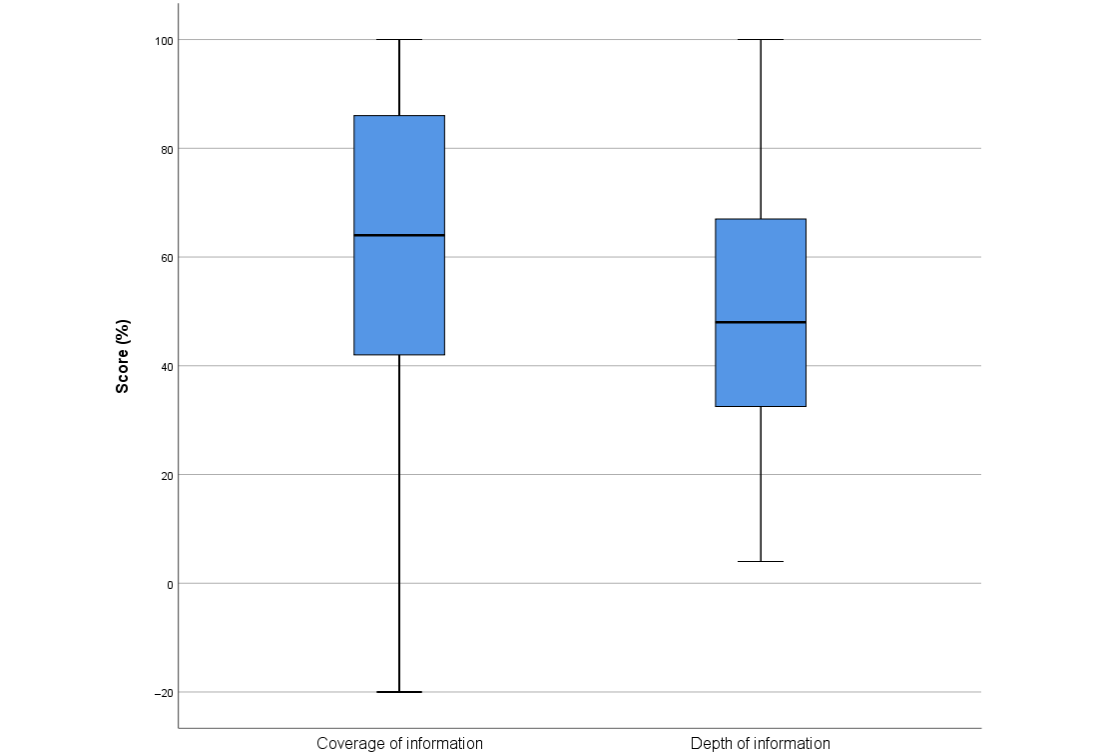
Quantitative evaluation of the coverage of information and the depth of information across all subtopics on infant feeding, introduction to solids, and physical activity in apps. A total of 59 evaluations were conducted for 47 apps. Median and IQR reported.

The coverage and depth of information provided by most apps were relatively poor (Table 1). Assessment with the content evaluation tool (Multimedia Appendix 3) found a median coverage, or breadth of subtopics correctly covered, of 64% (IQR 40%-87%; Figure 2). The depth, or completeness of information covered in the subtopics, had a median rating of 48% (IQR 32%-67%). The majority of app evaluations (31/59, 53%) had *low or no completeness* of subtopics (Tables 2 and 3). Furthermore, two-thirds of the app evaluations (38/59, 64%) showed that the apps had *poor* depth of information, and only 7% (4/59) of the app evaluations rated information as *complete* (Tables 2 and 3).

Detailed reporting of the coverage and depth of information within each subtopic are shown in Multimedia Appendix 4. Subtopics pertinent to clinicians are described below under *Coverage of Information in Subtopics* and *Depth of Information in Subtopics* and in Table 4.

**Table 1:** The quantitative coverage and depth of information based on Australian infant feeding and physical activity guidelines in all apps (N=59 evaluations of 47 apps).

Information quality		Median (%)	IQR (%)	Range (%)	Number of evaluations, n
**Coverage**					
	All apps	64	40-87	−20 to 100^a^	59
	Infant feeding apps	67	40-80	7 to 100	37
	Introduction to solid foods apps	50	0-88	−100 to 100^a^	37
	Infant activity apps	100	50-100	0 to 100	33
**Depth**					
	All apps	48	32-67	4 to 100	59
	Infant feeding apps	38	21-50	0 to 86	37
	Introduction to solid foods apps	38	6-50	0 to 100	37
	Infant activity apps	50	50-88	0 to 100	33

^a^Results with negative scores indicate apps with overall negative scoring for topics reported incorrectly.

**Table 2:** The qualitative evaluation of coverage of information based on Australian infant feeding and physical activity guidelines in all apps (N=59 evaluations of 47 apps).

Coverage of information	Poor, n (%)	Adequate, n (%)	Excellent, n (%)	Number of evaluations, n
All apps	38 (64)	8 (14)	13 (22)	59
Infant feeding apps	25 (68)	6 (16)	6 (16)	37
Introduction to solid foods apps	26 (70)	2 (5)	9 (24)	37
Infant activity apps	10 (29)	0 (0)	24 (71)	33

**Table 3:** The qualitative evaluation of depth of information based on Australian infant feeding and physical activity guidelines in all apps (N=59 evaluations of 47 apps).

Depth of information	Low or no completeness, n (%)	Partial completeness, n (%)	Complete, n (%)	Number of evaluations, n
All apps	31 (53)	24 (41)	4 (7)	59
Infant feeding apps	27 (73)	10 (27)	0 (0)	37
Introduction to solid foods apps	26 (70)	9 (24)	2 (5)	37
Infant activity apps	7 (21)	8 (56)	19 (24)	33

**Table 4:** The depth (completeness) of information in the subtopics reported (N=59 evaluations of 47 apps).

Completeness of information		Complete, n	Partially complete, n	Incorrect or incomplete, n	Number of evaluations, n^a^
**Encouraging and supporting breastfeeding**					
	Breastfeeding as the physiological norm	16	11	5	32
	Protection and promotion of breastfeeding	6	14	1	21
	Breastfeeding education for parents	12	12	1	25
**Initiating breastfeeding**					
	Physiology of breast milk and breastfeeding	4	17	1	22
	The first breastfeed	3	6	5	14
**Establishing and maintaining breastfeeding**					
	Difficulty establishing breastfeeding	1	10	2	13
	Factors affecting establishment of breastfeeding	1	10	5	16
	Monitoring an infant’s progress	8	14	4	26
	Maternal nutrition	4	11	7	22
**Breastfeeding, common problems, and their management**					
	Maternal factors affecting breastfeeding	3	15	1	19
	Infant factors affecting breastfeeding	1	12	3	16
**Expressing and storing breast milk**					
	Expressing breast milk	5	13	5	23
	Feeding with expressed breast milk	5	5	1	11
	Storage of expressed breast milk	10	5	9	24
**Breastfeeding in special situations**					
	Tobacco, alcohol, and other drugs	2	13	6	21
**Infant formula**					
	Preparing infant formula	2	4	7	13
	Using infant formula	6	6	4	16
	Special infant formula	1	5	0	6
**Introducing solids**					
	When should solid foods be introduced?	7	15	13	35
	What foods should be introduced?	3	3	21	27
	Foods and beverages most suitable for infants or foods that should be used in care	4	14	9	27
	Healthy foods in the first 12 months (continued exposure and opportunity to sample a wide variety of healthy foods)	10	9	5	24
**Infant activity**					
	Encouraging physical activity for infants from birth for healthy development	16	11	1	28
	Advice on types of infant physical activity and movements for development, including reaching and grasping; pulling and pushing; moving their head, body, and limbs during daily routines; and supervised floor play, including tummy time	10	13	2	25

^a^Not all apps included information on all subtopics.

#### Coverage of Information in Subtopics

Information coverage was lowest in apps related to infant feeding and introduction to solid foods. Infant activity subtopics were more likely to provide correct advice, with only 1 app reporting incorrect advice on encouraging infant activity for healthy development.

For information on infant feeding, incorrect advice was most frequently reported in the following subtopics: maternal nutrition during breastfeeding (3 apps), expressing and storing breast milk (3 apps across topics), breastfeeding in specific situations (tobacco, alcohol, and other drug use; 3 apps), and preparing and using infant formula (2-4 apps).

For information on introducing solid foods, incorrect advice was most frequently reported on the time to introduce solid foods (9 apps), first foods to introduce (15 apps), foods and beverages most suitable for infants (5 apps), and exposure to healthy foods for the first 12 months (3 apps).

#### Depth of Information in Subtopics

The ratings on the depth of information were lowest in apps related to infant feeding and introduction to solid foods (Table 4).

In apps on infant feeding, the depth of information was best reported for special infant formula, with 3 apps reporting *partially complete* information and 1 app reporting *complete* information. *Incomplete or incorrect* information was reported in all other subtopics across infant feeding apps. *Partially complete* information on infant feeding was more frequently reported than *complete* information.

In apps on introduction to solid foods, *incomplete or incorrect* information was reported in all subtopics. For appropriate first foods, *incomplete or incorrect* information was reported more frequently than *correct* information. *Partially complete* information on when to introduce solid foods and foods and beverages most suitable for infants was more frequently reported than *complete* information.

Most apps on infant activity reported *partially complete* information on encouraging infant activity for healthy development and *complete* information on the types of different infant physical activities or movements for development.

#### App Quality

**Figure 3:**
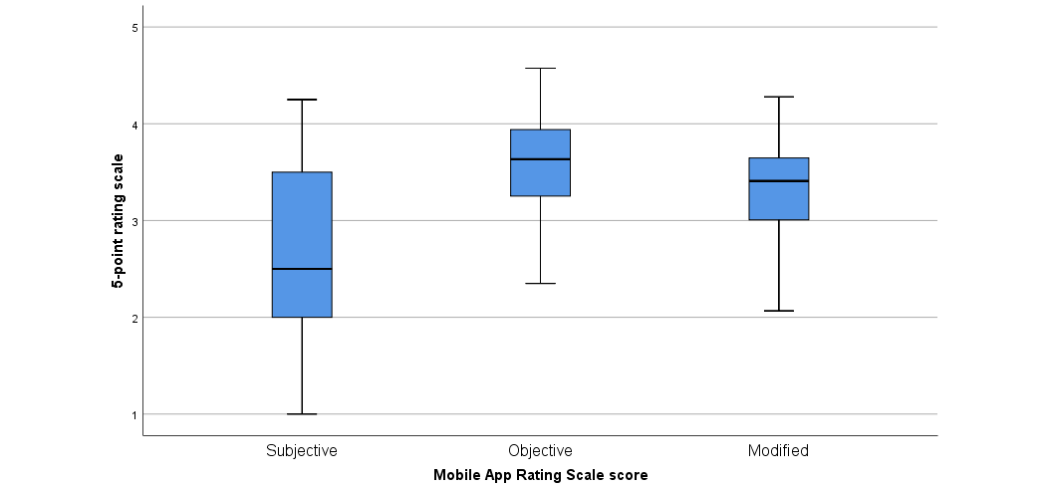
Quantitative evaluation of the 5-point Mobile App Rating Scale scores.

Table 5 and Figure 3 present the results of app quality evaluation using the MARS tool, which rates different objective and subjective dimensions of app quality.

**Table 5:** Mobile App Rating Scale quality ratings (N=59 evaluations of 47 apps).

MARS^a^ evaluation scores	Median	IQR	Range	Apps rated good, n	Apps rated excellent, n
Engagement subscale	3.00	2.60-3.40	1.80-4.20	12	0
Functionality subscale	4.25	3.75-4.75	2.0-5.0	26	24
Aesthetics subscale	4.33	4.0-4.67	2.0-5.0	32	10
Information quality subscale	3.60	3.0-3.80	1.75 -4.8	30	1
Objective MARS score^b^	3.63	3.24-3.99	2.07-4.28	38	1
Data security subscale	2.33	1.0-3.33	1.0-5.0	5	3
Accessibility subscale	3.00	2.0-3.67	1.0-5.0	18	1
Modified MARS score^c^	3.41	2.99-3.64	2.35-4.57	25	0
Subjective MARS score	2.50	2.0-3.5	1.0-4.25	20	1

^a^MARS: Mobile App Rating Scale.

^b^Objective MARS score=mean of engagement subscale+functionality subscale+aesthetics subscale+information quality subscale.

^c^Modified MARS score=mean of objective MARS score+data security subscale+accessibility subscale.

Overall, the quality of the apps was found to be mixed. Ratings were typically higher for items included in the objective score (especially the aesthetics and functionality subscales) than the subjective score. The inclusion of the 2 items on data security and accessibility lowered the median quality ratings of the apps, especially given that most apps rated poorly on data security (median 2.33; IQR 1.00-3.33).

**Figure 4:**
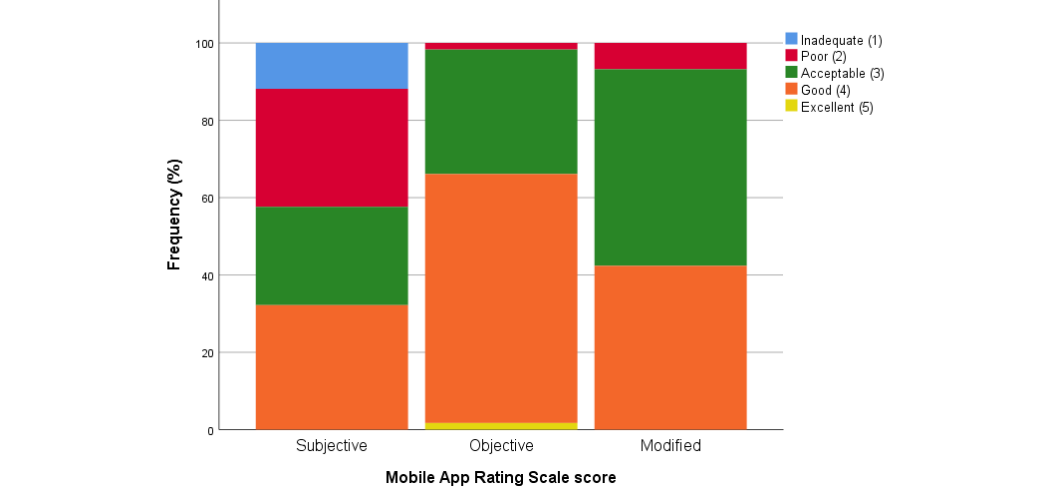
Qualitative evaluation of the 5-point Mobile App Rating Scale scores.

Figure 4 indicates that very few apps were rated *excellent* across all items on the MARS scales, although higher proportions were rated *good*, especially for the objective subscales.

On the subjective MARS scale, out of 47 apps, there were 21 apps (45%) that the reviewer reported they would never recommend to others or recommend to very few people. Furthermore, reviewers, who were child health clinicians or health researchers, reported that they would not pay to access 30 of the 47 apps (64%), although 4 of these were free, developed by the government or a community health organization.

A total of 31 apps reported the authors’ qualifications with health expertise, including doctors, nurses, lactation consultants, midwives, psychologists, physiotherapists, physical therapists, speech language therapists, health promotion officers, dietitians, nutritionists, occupational therapists, and sport therapists. As noted, 33 apps were developed by commercial entities, including 15 owned or developed by health care practitioners and 9 that consulted with health practitioners.

More detailed findings on the MARS and scores are reported in Multimedia Appendix 4.

We compared the MARS quality scores with the user-rated scores from the Apple App Store and Google Play Store reported in January 2019 (Multimedia Appendix 4). There was no significant correlation between the users’ app ratings and any of the MARS scores, but there was a significant correlation among the subjective, modified, and objective MARS scores (*P*<.001 each, 2-tailed).

#### Suitability of Infomation

Overall, 42% apps (25/59 evaluations) were rated *superior* for suitability of health information, 54% apps (32/59 evaluations) were rated as *adequate*, and 3% apps (2/59 evaluations) were *not suitable*. More detailed findings on the SAM scores are reported in Multimedia Appendix 4.

Few apps were rated *superior* on the cultural appropriateness items, although most apps were considered *adequate* on the cultural match for an Australian setting. A few apps were considered *superior* when they were suitable for an Australian setting and featured information for a non-Western culture and demography. Few apps presented information with representation of images and examples demonstrating cultural diversity. The 3 apps that contained culturally diverse images were all developed outside of Australia (but were available in English); the target populations were Croatian (*Baby Food Chart*), mainland Chinese and Hong Kong Chinese (*Info for Nursing Mum*), and Maori and Pacific Islander families (*Raising Children*).

Many apps provided instructions for taking clear and specific actions, with topics subdivided to motivate users—for example, in apps on introducing solid foods, information was subdivided into sections on the types of food to introduce, how to prepare food, how to feed infants, and how to encourage dietary variety. Most information was provided in a question-and-answer format, which was rated as *adequate* reader interaction.

#### Readability

The reading grade of app content was consistent across the tools used (Table 6).

There was a good correlation among reading grade scores using the 3 readability measures (*P*<.001; Multimedia Appendix 4).

**Table 6:** Readability scores of infant feeding and activity apps.

American grade level reading score	Median	IQR
Flesch-Kincaid score (online tool)	8	6-10
Flesch-Kincaid score (Microsoft Word)	8	6-9
Simple Measure of Gobbledygook score	8	6-9

However, very few apps met the Australian federal government’s recommended level for written health information of Grade 4 reading level or below: either 3 apps (using the F-K online tool), 4 apps (F-K in Word), or 5 apps (SMOG). A majority of apps met the South Australian government’s recommended level of Grade 8 level reading and below: 24 and 32 apps using F-K online tool and Microsoft Word tool, respectively, and 34 apps using the SMOG tool.

There was a low correlation among reading grades rated as not suitable, adequate, or superior in the SAM tool and the overall SAM score on adequacy of health information (*r*=0.25; *P*=.06).

#### Comparison With the 2015 Study

The 2015 study [4] used similar methods to evaluate infant feeding apps for parents. Although this study aimed to replicate it, some of the methods of analysis and presentation differed. Table 7 presents comparable findings between the 2 studies.

**Table 7:** Comparison of evaluation outcomes used in the original 2015 study and this study.

Instrument			This study (47 apps and 59 evaluations)	2015 study (46 apps and 46 evaluations)
**Content evaluation tool (%), median (IQR)**				
	Coverage of information		64 (40-87)	65 (58-71)
	Depth of information		48 (32-67)	Reported graphically
**App quality using Quality Component Scoring System (scored out of 100%)**				
	Median (IQR)		Not undertaken	49 (41-60)
	Proportion rated poor (<50% score)		Not undertaken	91
**App quality using Mobile App Rating Scale (scored out of 5 points)**				
	**Objective scale**			
		Median (IQR)	3.63 (3.24-3.99)	Not developed at the time of writing
		Proportion rated poor (%, ≤2.5 score)	2	Not developed at the time of writing
	**Modified scale**			
		Median (IQR)	3.41 (2.99-3.64)	Not developed at the time of writing
		Proportion rated poor (%, ≤2.5 score)	7	Not developed at the time of writing
	**Subjective scale**			
		Median (IQR)	2.50 (2.0-3.5)	Not developed at the time of writing
		Proportion rated poor (%, ≤2.5 score)	54	Not developed at the time of writing
**Suitability Assessment of Material (%)**				
	Superior overall (70%-100%)		44	15
	Adequate overall (40%-69%)		53	39
	Not suitable (0%-39%)		3	42
**Readability, median (IQR)**				
	Flesch-Kincaid online tool		8 (6-10)	8 (7-10)
	Flesch-Kincaid Word tool		8 (6-9)	8 (7-10)
	Simple Measure of Gobbledygook		8 (6-9)	7 (7-8)

## Discussion

### Principal Findings

This study evaluated 47 apps on infant feeding and physical activity and found that the information content within these apps was largely poor for coverage and depth of information presented. This study updated a systematic assessment of 46 apps available from 2013 to 2014, which similarly found poor quality information for parents [4]. Although the quality of apps, rated through the MARS, improved since the previous study, the credibility of advice did not improve; of the 59 evaluations, almost two-thirds reported *poor* coverage of information, and over half the app evaluations reported *incorrect or incomplete information* on the topics addressed.

Reviewers identified information contrary to the Australian guidelines on infant feeding [40] and physical activity [41] in 21 apps. Many of these apps were developed outside of Australia, that is, in America, the United Kingdom, and the European Union, where the official guidelines may be different from those in Australia. Although 1 app with incorrect information was developed in Australia and involved health care professionals, it was developed by a medical device company whose promotion of its breast pumps likely interfered with the provision of correct breastfeeding information.

The results using the MARS tool were largely positive. In this systematic assessment, the apps were rated as moderately engaging, functional, and visually appealing, and these apps clearly reported whether the information came from credible sources. One strength of the MARS tool is that it provides a multidimensional overview of app characteristics, all of which contribute to the user’s experience of an app and the capacity to learn from it. In this case, most apps rated well on engagement, functionality, and aesthetics. However, in MARS, the quality and quantity of the information content constitute only 2 out of the 7 domains that contribute to the final score. Therefore, the information items were inadequate to reflect the importance of the content in this context. In other words, the MARS tool gave relatively little weight to the quality and accuracy of the information provided to consumers about infant nutrition and activity.

Therefore, although many apps were qualitatively evaluated as *good* to *excellent* on the objective and modified MARS score, the information contained within these apps was poor. This is reflected in the subjective scores for the apps, with a median of 2.50 and most apps falling between *inadequate* and *acceptable*. Reviewers reported that nearly half of all apps were *poor* or *inadequate* for recommending to others.

Brown et al [55,56] systematically reviewed the content of pregnancy apps against the Australian national recommendations on healthy eating for pregnancy. Similar to this study, they found that the apps were of moderate quality (mean objective MARS score was 3.05 [SD 0.66] and 3.52 [SD 0.58] for iOS and Android apps, respectively), with the functionality scale (mean 3.32 [SD 0.66]and 4.06 [SD 0.67], for iOS and Android apps, respectively) rated highest. These reviews also found incorrect and potentially harmful information in 3 apps about avoiding alcohol during pregnancy, fasting, restricting dairy products, and using dairy alternatives to meet calcium requirements.

Similarly, a review by Richardson et al [33] on apps for parents of infants in neonatal intensive care units highlighted the inconsistencies in quality ratings according to the method used. They identified a discrepancy between high app quality ratings using the MARS tool and those using the Trust It or Trash It tool [57] for evaluating clearly reported sources of health information.

Although most apps were rated suitable for their potential users on many dimensions, they scored less on readability and cultural appropriateness. Using the readability tools, only a handful of apps met the Australian government’s requirement of year 4 reading level (3-5 apps, depending on what readability tool was used), although a majority met the less stringent South Australian government’s requirement of year 8 level. However, the reviewers’ SAM ratings suggested that most apps used a suitable vocabulary and appropriate style and ordering.

### Limitations

The app market is highly dynamic, and this study captured a cross-sectional snapshot of infant feeding and play apps available at one time point. Several apps in the previous study [4] were not available for download in 2018 and 2019. Even in this study, some apps had been removed from the marketplace by the time of writing, demonstrating the volatility of this information source. This changing availability makes the process of systematically reviewing apps challenging and potentially limited if key resources are not available during the selection phase.

Changes in the app search engines impacted the search process. The removal of the iOS App Store feature on iTunes in September 2017 [37] meant that the reviewers could only search using an iPhone instead of a desktop computer and prevented reliable double-checking of apps found for screening. As with other mHealth reviews conducted after September 2017 [56], double-checking of apps available in the App Store was conducted on Fnd, a web-based search tool [58], which reduces the flexibility of consumers to search for apps on desktop and synchronize the app download to their smartphone.

Search optimization, which refers to optimizing smartphone apps for visibility in search engines and browsing [59], also affected the types of apps found. To reflect the external validity of apps found by users searching through a smartphone app search engine, the authors did not include handsearching or searching for apps outside of the app search engine. Oversaturation of search engines with apps that are malware, spam, counterfeit, or contain copyrighted and farmed content [60-62] results in excessive number of apps; over 5000 apps were found during screening (Figure 1). This affects the apps found through Google Play, as search results are limited to 250 apps per search; therefore, if irrelevant apps are found in keyword searches and listed in the first 250 apps found, this is a nonoptimal search strategy, and relevant apps will be displaced from the search and not included in the analysis. This excessive number of apps caused relevant apps to be displaced by irrelevant apps; for example, a nongovernment organization–developed app on tummy time (*Red Nose* *Safe Sleeping*) was available on both search engines, but poor search optimization for keywords resulted in the app not being found through Google Play searches.

This study used keywords in English and evaluated apps available with English as the main language option. We acknowledge the multicultural background of the Australian population and the need for apps suitable for culturally and linguistically diverse parents [63], and a limitation of our study may exclude app users without English language proficiency (approximately 3.5% of Australians aged 15-49 years in 2016, data from Australian Bureau of Statistics [64]). Of the 102 relevant apps screened (Figure 1), only 3 were excluded for not being available in English. It is likely, however, that non-English–speaking parents may search for non-English sites. Zhao et al [32] used the 360 App Store and Chinese language keywords to conduct similar research, focusing on apps used by non-English–speaking parents. This shows the potential for this approach to include different app marketplaces and language search terms to explore apps available for different groups of parents.

### Interrater Reliability

The IRR results (Multimedia Appendix 4) showed considerable discrepancies on some instruments. There was low agreement on readability scores, which may reflect the different passages of text selected for evaluation by the pairs of reviewers. This is likely as reading grade level was calculated using objective tools, rather than individual reviewer assessment.

Although there was acceptable agreement on the modified MARS scores between the pairs of reviewers, there was far less agreement on the subjective MARS items. This difference in scoring may reflect the reviewer’s perspectives (a dietitian researcher’s evaluation compared with a child and family health nurse clinician’s evaluation), affecting their likelihood of recommending an app and also their evaluation of its suitability for consumers using the SAM tool.

There was also mixed assessment on the coverage and depth of information on infant feeding, introduction to solids, and infant activity subtopics. Although reviewers evaluated content against the same government guidelines, they may have varied with regard to stringency in certain subtopics, such as deciding whether infants with appropriate signs of readiness can start solid foods between 4 and 6 months or if exclusive breastfeeding for 6 months was imperative [65]. Similarly, reviewers varied on how much information constituted *complete* or *partially complete* advice, affecting IRR.

### Comparison With Prior Work

In replicating an earlier study of infant feeding and activity apps, we were able to compare the quality and content of apps available during 2018 to 2019 and those available 5 years earlier. The increased access to smartphones and the utilization of app-based health information among the Australian population over that time suggest that this study is highly relevant and timely. However, notwithstanding the increased suitability and functionality of these apps (measured with the SAM and MARS tools, respectively), the quality and accuracy of much of the information in apps have not greatly improved over time.

Since the 2015 study, this study identified growth in apps that are from reputable sources such as the government, universities, and health professionals: only 2 apps with university endorsements were found in the previous study [4] compared with 14 apps with university or government development or affiliation in this study. In addition, 3 apps were also used in research [52-54]. This finding indicates a positive transition of trustworthy sources that leverage the increased usage of technology and offer credible information to a wider population. This study also indicated that 9 apps were available in languages other than English, compared with none in the previous study. This demonstrates improved multilingual resources for a growing culturally diverse population.

This study was more comprehensive in that it looked at smartphone apps in Android and iOS, both paid and free to access. However, the previous study also looked at websites available on this topic, which might still be a widely used tool given the widespread use of *Dr Google* to search for pregnancy, birthing, and parenting information, which may not be accurate, credible, reliable, or safe [3,13,66-68].

Despite an increased proportion of apps published by reputable sources, this study also found relatively few good quality apps available. We reiterate the earlier recommendation [5] to establish a certified endorsement for apps similar to the Health on the Net Foundation Code of Conduct used on the website. This code of conduct is used to standardize the reliability of medical and health information available on the World Wide Web [69] and encourages website developers to maintain the quality standards of the organization. The 2015 study found that websites that subscribed to this code of conduct certificate had higher quality scores [4].

Only 2 apps (*WebMD Baby* and *Pregnancy and Baby Tracker* [formerly *What to Expect*]) were included across both studies, highlighting that the app marketplace changes continuously. A potential reason for the short *shelf life* of some apps could be the high maintenance cost required to keep the apps updated with the evolving smartphone operating systems. Intervention studies of health apps have reported technical issues with the implementation of their app [70], such as updates in the operating system or app impeding participant access [71-74]. This indicates that app functioning requires ongoing maintenance. This might be a challenge for apps that are developed with limited funding from universities or governments compared with commercial companies that often have higher budgets. Further research is required to explore factors that impact the shelf life of health-related apps and to develop suggestions on sustainable ways to leverage technology to share health-related information.

The use of the MARS tool, specifically developed for app evaluation, is a strength of this study. However, the MARS tool had not been published at the time of the 2015 study [4]. The original study adapted the Quality Component Scoring System to assess apps; however, this tool was originally developed to evaluate the quality of medical websites [75], and not all items were appropriate for apps.

Although the national infant feeding guidelines in Australia have remained consistent since the previous study [40], the uptake of this information has not improved. Research indicates that gaps exist in the current infant feeding practices. Begley et al’s [76] research with mothers, using focus groups, in Western Australia found that less than half of the participants had heard of the Australian Infant Feeding Guidelines or were aware that the recommended age for the introduction of solid foods was around 6 months; many participants believed that the guidelines were based on opinion rather than scientific research. A survey of mother-infant dyads in Western Australia and South Australia during 2010 to 2011 found that the feeding behaviors of participants fell short of Australian feeding guidelines, where although 93% of mothers initiated breastfeeding, only 42% of infants were breastfed to 6 months, and 97% of infants received solid food by 6 months [77].

### Implications for Practice

It is well established that early childhood experiences have a significant impact on optimal child development [40]. Child and family health nurses in the community play an integral role in monitoring children’s growth and development and providing guidance and support to parents. Child and family health nurses and lactation consultants work in an environment that has limited staffing and financial resources.

Many parents live in isolation from extended families and turn to social networking sites for advice from peers for health-related information. Conflicting advice and lack of continuity of care from health professionals often adds to their confusion about how they should care for their children [78,79]. Increasingly, parents require direction and guidance to seek evidence-based educational resources. Parents seek information that is easily accessible and affordable. Information provided during consultation in the child and family health nurse clinics often dispels the parents’ concerns, but parents may be unable to absorb all the information at one time and often require educational resources that they can refer back to once they have gone home. Appropriate internet websites and smartphone apps are key to meeting this need.

Parents, particularly first-time parents, are bombarded with information from a variety of sources, especially from websites and smartphone apps. Although information evaluation tools supported by librarians, academics, and the government [57,80,81] for critically assessing health information quality can support the health and digital literacy of parents, these tools may not be widely known to the layperson. Similarly, clinicians who regularly support new parents often receive requests for advice about apps and cannot confidently provide a recommendation if they are unaware of app evaluation tools [82] or have insufficient time to evaluate apps. One way to overcome this challenge is to establish a *trusted app* or similar logo (logo similar to that of the Health on the Net Foundation Code of Conduct’s logo) or a repository of approved apps, such as the United Kingdom’s National Health Service Apps Library [83], which can be applied to evidence-based apps that do not promote particular products.

### Conclusions

Improved functionality, suitability, and user engagement of smartphone apps in recent years are welcome developments for parents seeking guidance on infant feeding and activity. However, the high-quality content of apps is critical to good health outcomes. Assessment of available apps revealed that some provided very useful information, but there was wide disparity in reliability and consistency with evidence-based knowledge. Many apps provided additional information outside their focal topic or area of expertise; others offered information largely designed to promote their products. In some instances, this information was limited and did not provide comprehensive advice consistent with evidence-based guidelines.

## Appendix

Multimedia Appendix 1 Google Trends for app search key terms.

Multimedia Appendix 2 App search and evaluation protocol.

Multimedia Appendix 3 App evaluation tools used.

Multimedia Appendix 4 Supplementary tables and figures.

## Abbreviations

F-K: Flesch-Kincaid
iOS: iPhone Operating System
IRR: interrater reliability
MARS: Mobile App Rating Scale
mHealth: mobile health
SAM: Suitability Assessment of Materials
SMOG: Simple Measure of Gobbledygook
