# Supplementary material for: Content and Quality of Infant Feeding Smartphone Apps: Five-Year Update on a Systematic Search and Evaluation
Source: JMIR Mhealth Uhealth. 2020 May 27;8(5):e17300. doi: 10.2196/17300 (PMC7287747; doi:10.2196/17300)
Supplement: Multimedia Appendix 1 [file mhealth_v8i5e17300_app1.docx]

Interest over time: Numbers represent search interest relative to the highest point on the chart for the given region and time. A value of 100 is the peak popularity for the term. A value of 50 means that the term is half as popular. A score of 0 means there was not enough data for this term.

Figure 1: Interest of ‘breastfeeding app’ as worldwide search term in the Google search engine, January 2013 to January 2019.

Interest over time: Numbers represent search interest relative to the highest point on the chart for the given region and time. A value of 100 is the peak popularity for the term. A value of 50 means that the term is half as popular. A score of 0 means there was not enough data for this term.

Figure 2: Interest of ‘baby feeding app’ as worldwide search term in the Google search engine, January 2013 to January 2019, worldwide search.

Interest over time: Numbers represent search interest relative to the highest point on the chart for the given region and time. A value of 100 is the peak popularity for the term. A value of 50 means that the term is half as popular. A score of 0 means there was not enough data for this term.

Figure 3: Interest of ‘baby care app’ as worldwide search term in the Google search engine, January 2013 to January 2019, worldwide search.

Interest over time: Numbers represent search interest relative to the highest point on the chart for the given region and time. A value of 100 is the peak popularity for the term. A value of 50 means that the term is half as popular. A score of 0 means there was not enough data for this term.

Figure 4: Interest of ‘parenting app’ as worldwide search term in the Google search engine, January 2013 to January 2019, worldwide search.

Interest over time: Numbers represent search interest relative to the highest point on the chart for the given region and time. A value of 100 is the peak popularity for the term. A value of 50 means that the term is half as popular. A score of 0 means there was not enough data for this term.

Figure 5: Interest of ‘baby food app’ as worldwide search term in the Google search engine, January 2013 to January 2019, worldwide search.

Interest over time: Numbers represent search interest relative to the highest point on the chart for the given region and time. A value of 100 is the peak popularity for the term. A value of 50 means that the term is half as popular. A score of 0 means there was not enough data for this term.

Figure 6: Interest of ‘baby formula app’ as worldwide search term in the Google search engine, January 2013 to January 2019, worldwide search.

Interest over time: Numbers represent search interest relative to the highest point on the chart for the given region and time. A value of 100 is the peak popularity for the term. A value of 50 means that the term is half as popular. A score of 0 means there was not enough data for this term.

Figure 7: Interest of ‘baby play app’ as worldwide search term in the Google search engine, January 2013 to January 2019, worldwide search.

Interest over time: Numbers represent search interest relative to the highest point on the chart for the given region and time. A value of 100 is the peak popularity for the term. A value of 50 means that the term is half as popular. A score of 0 means there was not enough data for this term.

Figure 8: Interest of ‘baby development app’ as worldwide search term in the Google search engine, January 2013 to January 2019, worldwide search.
