# Supplementary material for: Content and Quality of Infant Feeding Smartphone Apps: Five-Year Update on a Systematic Search and Evaluation
Source: JMIR Mhealth Uhealth. 2020 May 27;8(5):e17300. doi: 10.2196/17300 (PMC7287747; doi:10.2196/17300)
Supplement: Multimedia Appendix 2 [file mhealth_v8i5e17300_app2.docx]

# Protocol for scientific audit: An audit of smartphone applications for advice on infant feeding & growth

# Review question

The objective of this systematic analysis is to critically evaluate smartphone applications aimed at assisting parents with infant feeding practices, promoting healthy infant growth and infant movement.

| **Summary** | |
| --- | --- |
| Targeted population | Applications with content targeted at parents with young infants (≤1 year) |
| Applications | Applications aimed at providing parents with information/ resources on one of the following:   1. Healthy milk feeding behaviours (breastfeeding, formula   feeding, bottle feeding, feeding to appetite)   1. Healthy solid food feeding behaviours (age of introduction, types of foods introduced, repeated exposure, varied exposure, reducing exposure to unhealthy food/beverages, feeding to appetite) 2. Promoting free movement (reducing restraint, allowing natural movement). |
| Timeframe of last updated websites and apps | 2008 *(Apps were developed)* |

# Application selection:

## Smartphone application software

For the purpose of this study, two search engines were used to search mobile smartphone applications: App Store for iOS and Google Play for Android.

## Inclusion and exclusion criteria

### Included:

- English language, either as primary language or English language option
- Free and paid applications intended for parents/caregivers assisting in:
  - Healthy infant feeding (breastfeeding, bottle feeding, introduction to solids, other feeding behaviours related to solid foods)
  - Physical activity (freedom of movement/tummy time)

### Excluded:

- Non-English language
- Aimed at children greater than 1 years of age
- Applications that could not be accessed due to app being broken or other app errors
- Apps that contain the same content as electronic books, news, magazines, podcasts, blogs or Word documents, in a smartphone app format
- Games/gaming apps, indicated by cartoon icons or ‘game’ used in app name or developer name
- Non-relevant apps found in search engines due to algorithm errors
- Tracking, timing, recording, chart or calendar-**only** apps without other information about healthy infant feeding and physical activity

## Search Terms

### Table 1. Target behaviours aimed to identify in applications and key words related to the behaviours

| **General key terms**  ***Do not use “” or ‘’ marks during the app store searches*** |
| --- |
| SET 1: Healthy milk feeding behaviours (breast, formula bottle)  **“Infant feeding”** |
| 1. Infant feeding |
| 1. Baby feeding |
| 1. Breast feeding |
| 1. Formula feeding |
| 1. Bottle feeding |
| SET 2: Healthy solid food feeding behaviours (age of introduction, types of foods introduced, repeated exposure, varied exposure, reducing exposure to unhealthy food/beverages)  **“Feeding solids”** |
| 1. Baby solids |
| 1. Baby food |
| 1. Baby weaning |
| SET 3: Promoting free movement (reducing restraint, allowing natural movement).  **“Infant activity”** |
| 1. Infant activity |
| 1. Tummy time |
| 1. Baby exercise |

Search for each term individually.

Screen and analyse the content of each search separately.

## Selection criteria

The selection criteria were developed to suit the content and aim of a project being conducted by a research team on infant feeding behaviours. This will aid in identifying reliable apps which Australian consumers (parents/caregivers) may possibly use. Hence, the intended audience, country of origin and variable of interest have been synced with the larger project.

### Table 2. Selection criteria for application analysis

| **Selection criteria** | **Include** | **Exclude** |
| --- | --- | --- |
| Application content | Any type of smartphone app, free or paid | - Electronic books, news, magazines, blogs, podcasts and word documents in smartphone app format |
| Intended audience | - Parents or primary caregivers with information on infants and children, 0 to 1 years - English language, as primary language or language option | - Information on older children, ≥1 years - Non-English language - Machine translation of non-English app to English^1^ |
| Year of development or last update | - 2008 | N/A |
| Variables of interest | Application must report on:   1. Healthy milk feeding behaviours   *Healthy milk feeding behaviours = breast, formula bottle, expressed breast milk, frequency, timing, correct preparation, feeding on demand, non-nutritive feeding*   1. Healthy solid food feeding behaviours   *Healthy solid food feeding behaviours = age of introduction, types of foods introduced, repeated exposure, varied exposure, reducing exposure to unhealthy food/beverages*   1. Promoting free movement (reducing restraint, allowing natural movement)   *Promoting free movement = reducing restraint, allowing natural movement, child activity, physical activity, sedentary behaviours (TV viewing, screen time)* | Exclude apps not reporting on any of the topics. This includes:   - Games/gaming apps, indicated by cartoon icons or ‘game’ used in app name or developer name - Non-relevant apps found in search engines due to algorithm errors - Tracking, timing, recording, chart or calendar-**only** apps without other information about healthy infant feeding and physical activity   Exclude apps that are malware, spam, counterfeits, copycats or contain copyrighted content:   - Apps with stolen content from other apps or websites^2^ - Apps with farmed content^3^ |

^1^ Machine translation refers to “use of software to translate text or speech from one language to another” (<https://en.wikipedia.org/wiki/Machine_translation>), and will be identified by poor readability of app content in English.

^2^ Stolen content from apps or websites refers to information plagiarised or duplicated from original sources.

^3^ Content farming describes a “website or network of sites that publishes large amounts of inexpensive, often low-quality content specifically designed to generate search engine traffic and advertising revenue” (<http://www.econtentmag.com/Articles/Resources/Defining-EContent/What-is-a-Content-Farm-78370.htm>).

Apps with farmed content may contain malware that can hack user’s phones, financial details and personal details: <https://thenextweb.com/apple/2010/07/05/app-store-app-farm-steal-your-money/>

An example of stolen content is below:

| **Original content** | **Stolen content, highlighted in red** |
| --- | --- |
| App and website: Annabel Karmel <https://www.annabelkarmel.com/au/recipes/no-cook-purees/>  © 2019 Annabel Karmel Group Holdings Limited  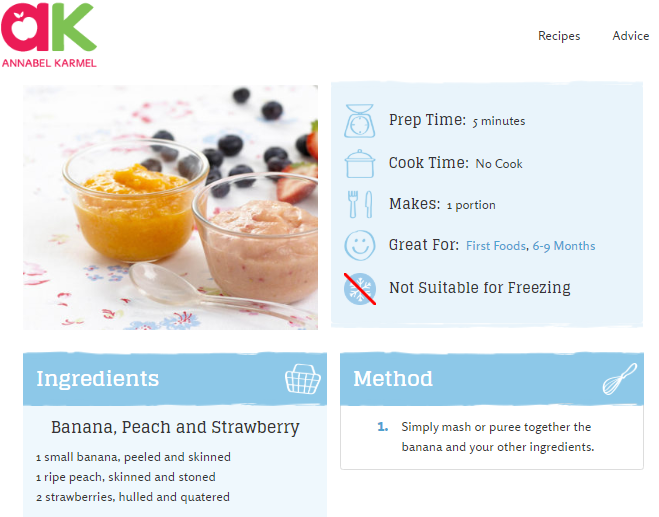 | App: Baby Food Recipes  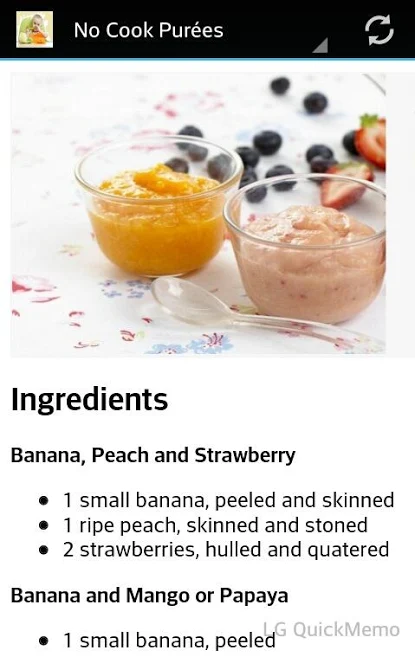 |
| Website: HealthED New Zealand  <https://www.healthed.govt.nz/resource/eating-healthy-babies-and-toddlersng%C4%81-kai-t%C5%8Dtika-m%C5%8D-te-hunga-k%C5%8Dhungahunga>  © 2011 Ministry of Health – Manatū Hauora  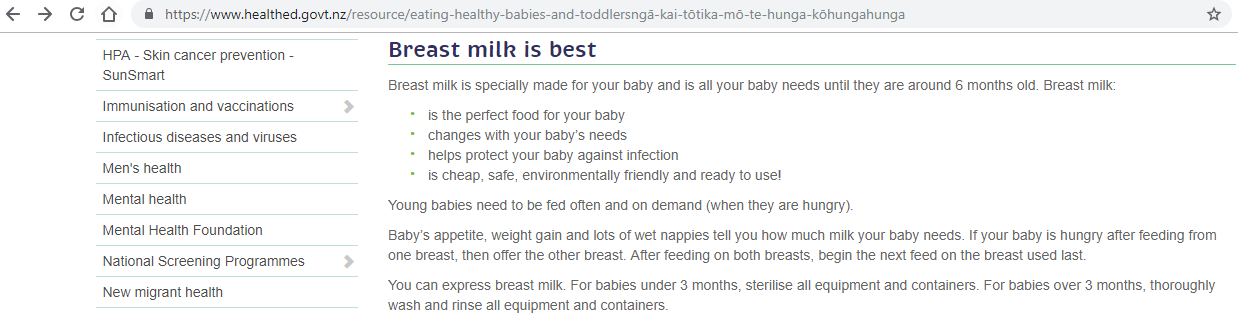 | App: Healthy Nutrition Guide Babies  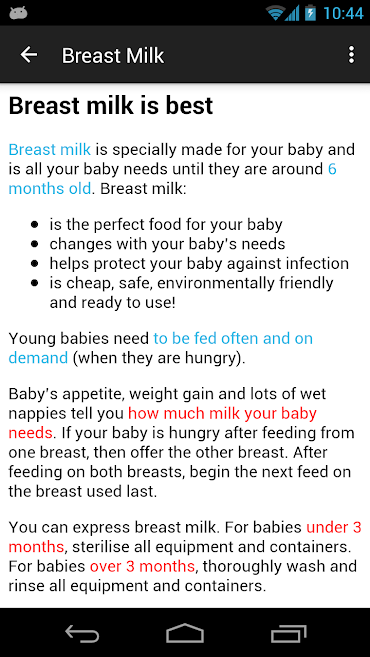 |
| Website: BabyCenter Australia  <https://www.babycenter.com.au/a8792/how-to-breastfeed-a-visual-guide>  © 1997-2019 BabyCenter, L.L.C.  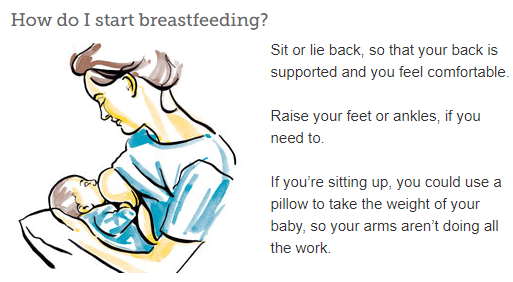 | App: How to Breastfeed  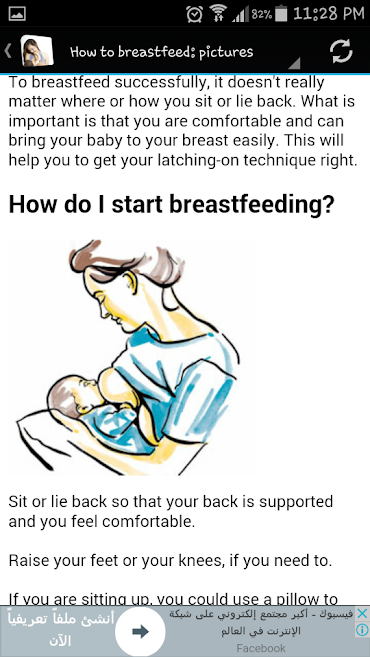 |

An example of machine translated content is below:

| **App content** | **Poor readability in English** |
| --- | --- |
| 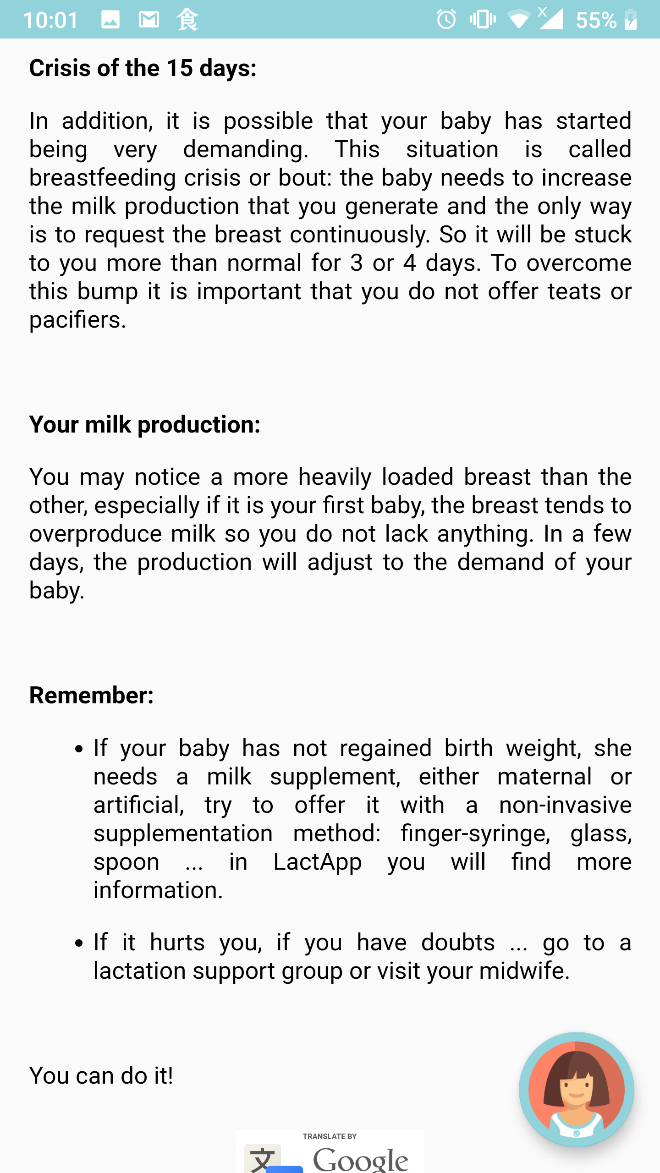 | “… the baby needs to increase the milk production that you generate and the only way is to request the breast continuously” – refers to the positive feedback loop of breastfeeding, but this is unclear to the consumer. |
| 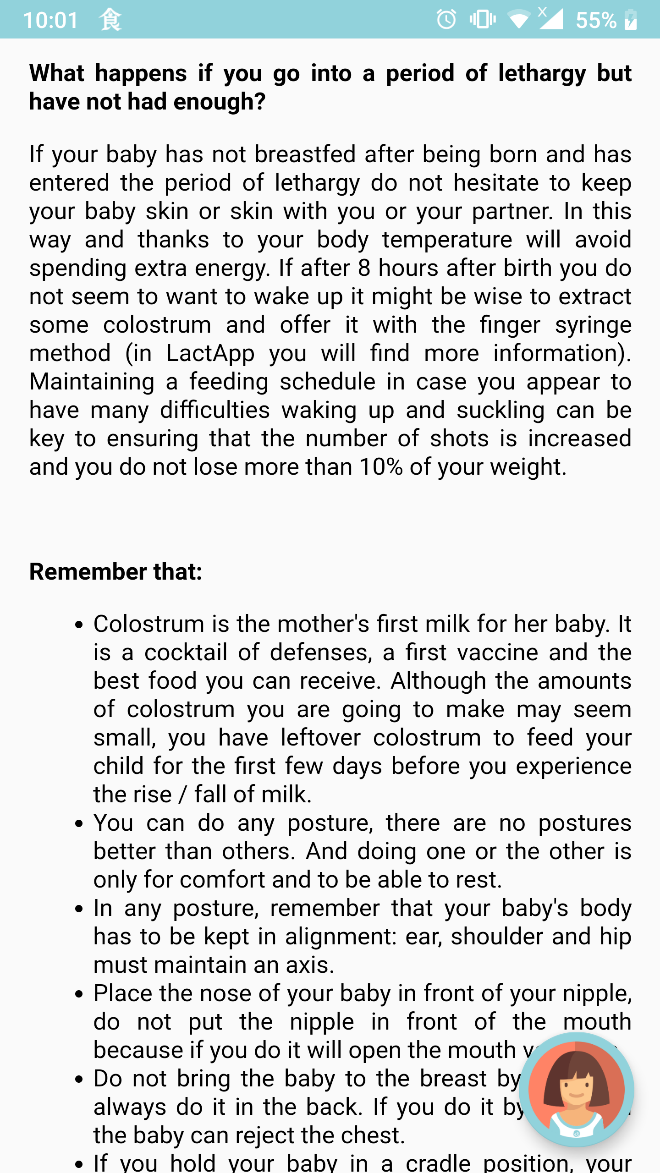 | “What happens if you go into a period of lethargy but have not had enough?” – refers to maternal tiredness interfering with breastfeeding, but this has poor comprehension to an English-language consumer. |
| 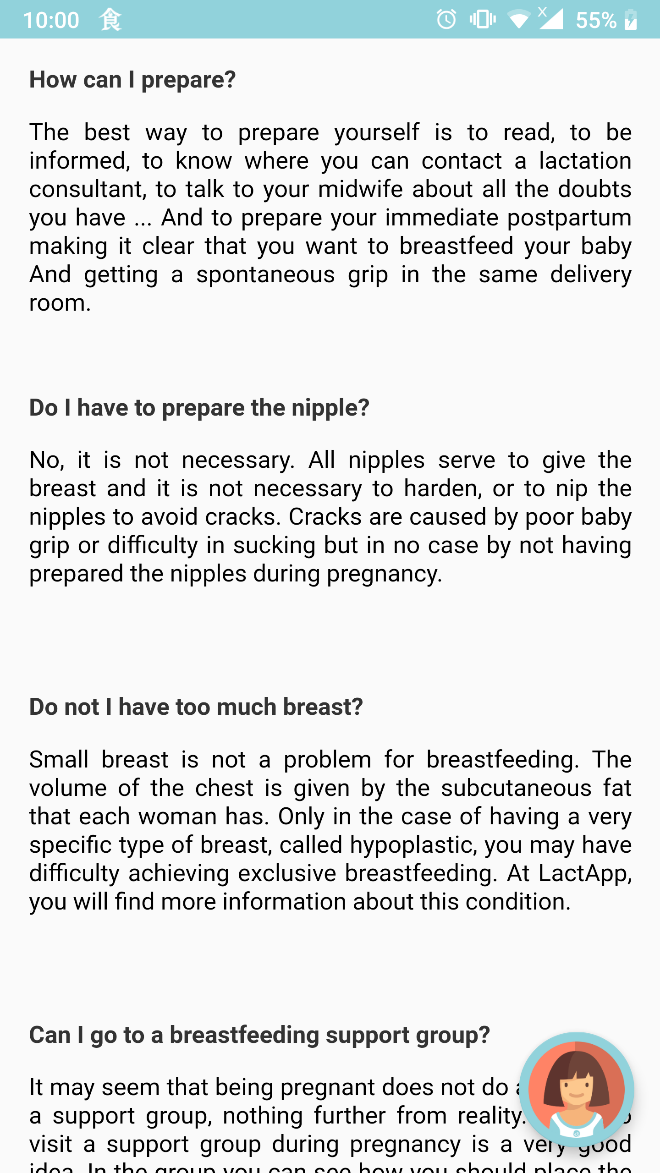 | “Do not I have too much breast?” – refers to maternal breast size and complications associated with breastfeeding, but this also has poor comprehension to an English-language consumer. |

An example of apps with farmed content is below:

| **App with farmed content in preview** | **Indication of poor app quality** | **Indication of content farming developer** |
| --- | --- | --- |
| 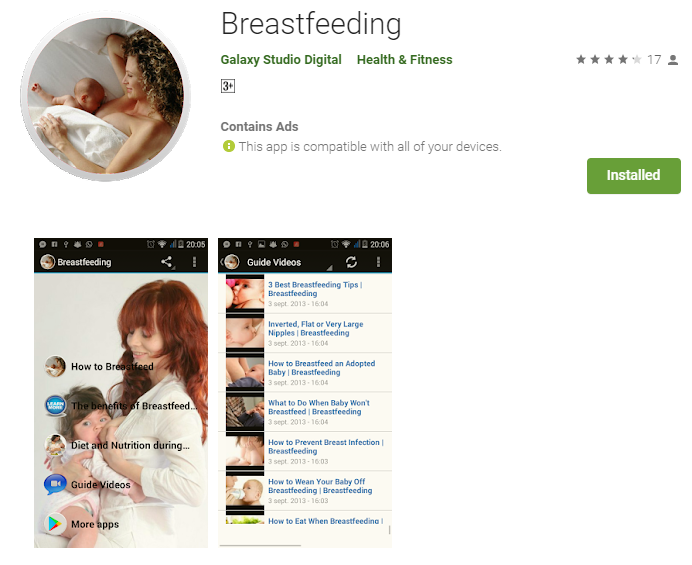App: Breastfeeding | Description of app is poor:  “A mother who nurses her baby - this is one of the most beautiful images God can create. It is also one of the simplest. Breastfeeding is so natural, in fact, that we have been doing it for millions of years. (Indeed, without it, the human race would not have survived.)”  App reviews: this app has 17 ratings and one only one review of poor quality, which can indicate that the review was purchased or generated by a bot.  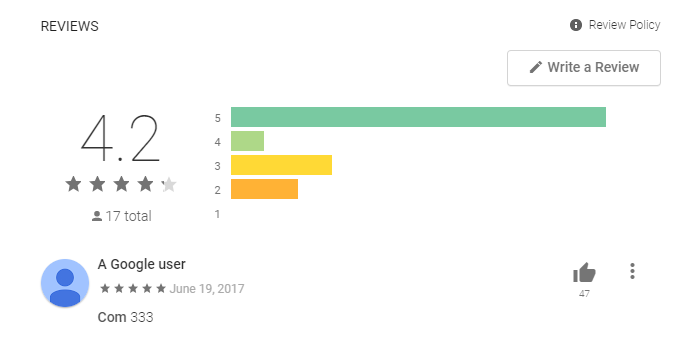 | Developer: Galaxy Studio Digital  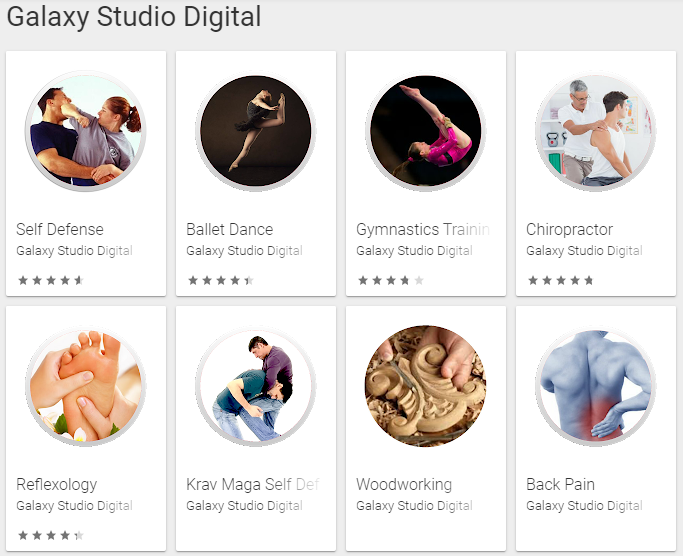Developer has developed a variety of unrelated apps of similar quality |
| App: Breastfeeding - Stories/Videos  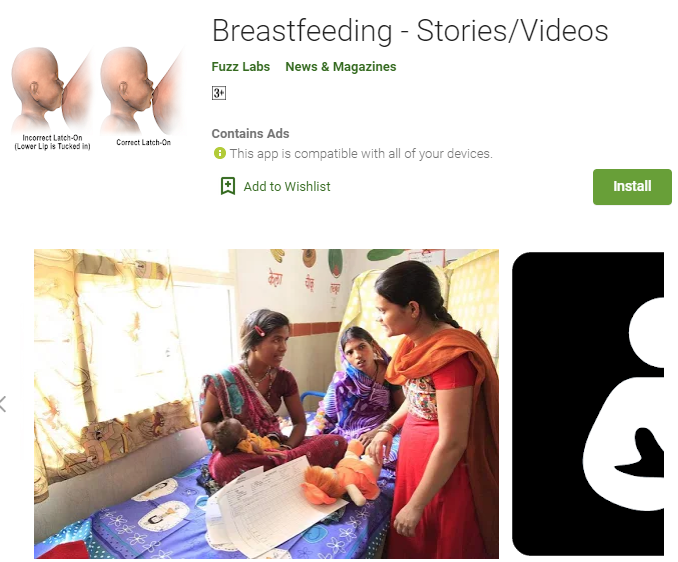 | Description of app is poor:  “Breastfeeding is an important aspect of motherhood. See how everyday multitudes of people around the world are dealing with it.”  App preview is poor and displays a scientific diagram, a photograph and a logo, indicating that this content may be randomly generated and fraudulent. | Developer: Fuzz Labs  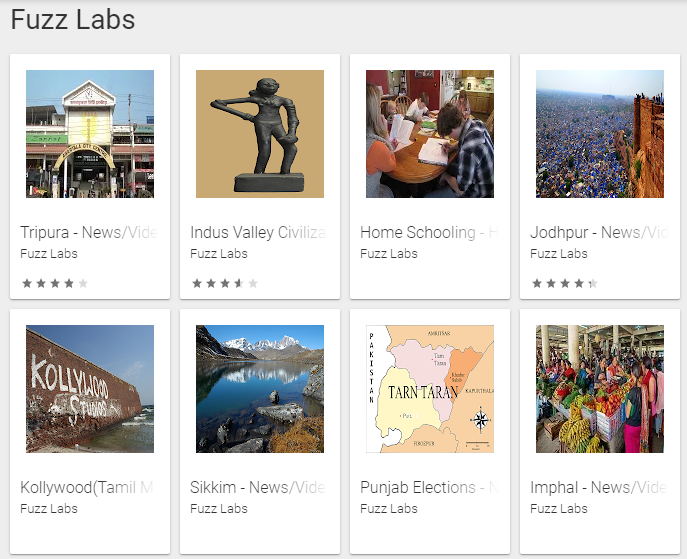Developer has developed a variety of unrelated apps of similar quality |
| App: Baby First Foods  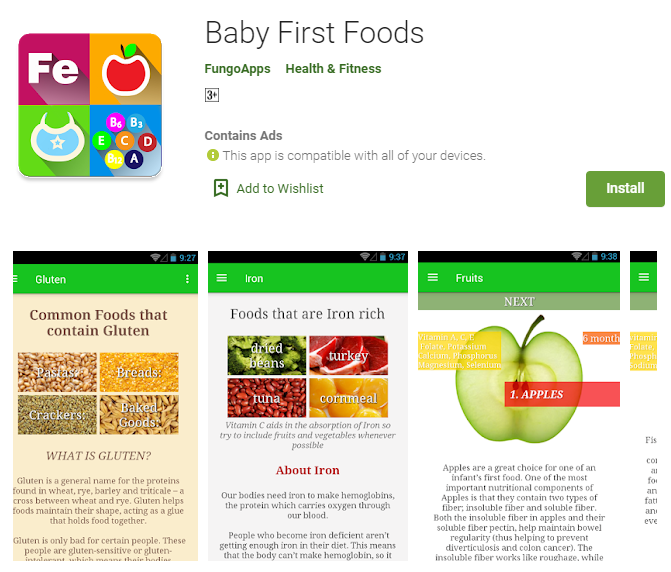 | Description of app does not adequately and coherently describe its content:  “- First Food  - Solid Food  - Common Foods that contain Gluten  - Foods that are Iron rich  - Most and least likely to induce an allergic reaction  - About Pesticides” | Developer: FungoApps  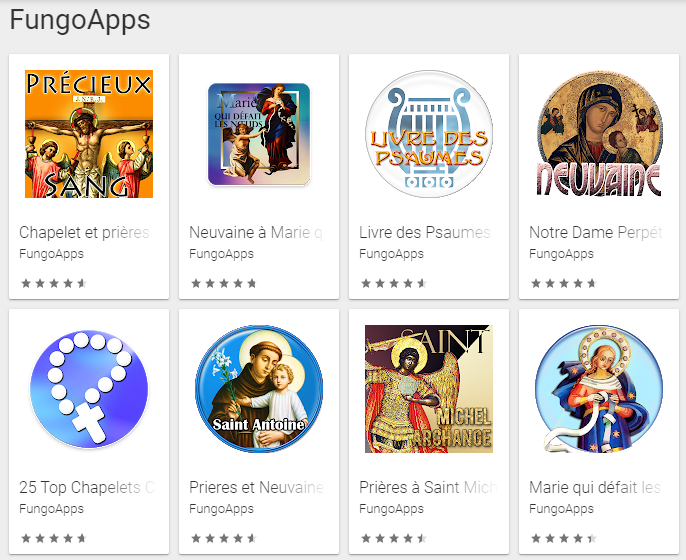Developer has developed a variety of unrelated apps of similar quality |

Example of Google Play app search, with relevant and non-relevant apps selected.

e.g. ‘baby food’ search on Google Play, Apps section, Free apps only.


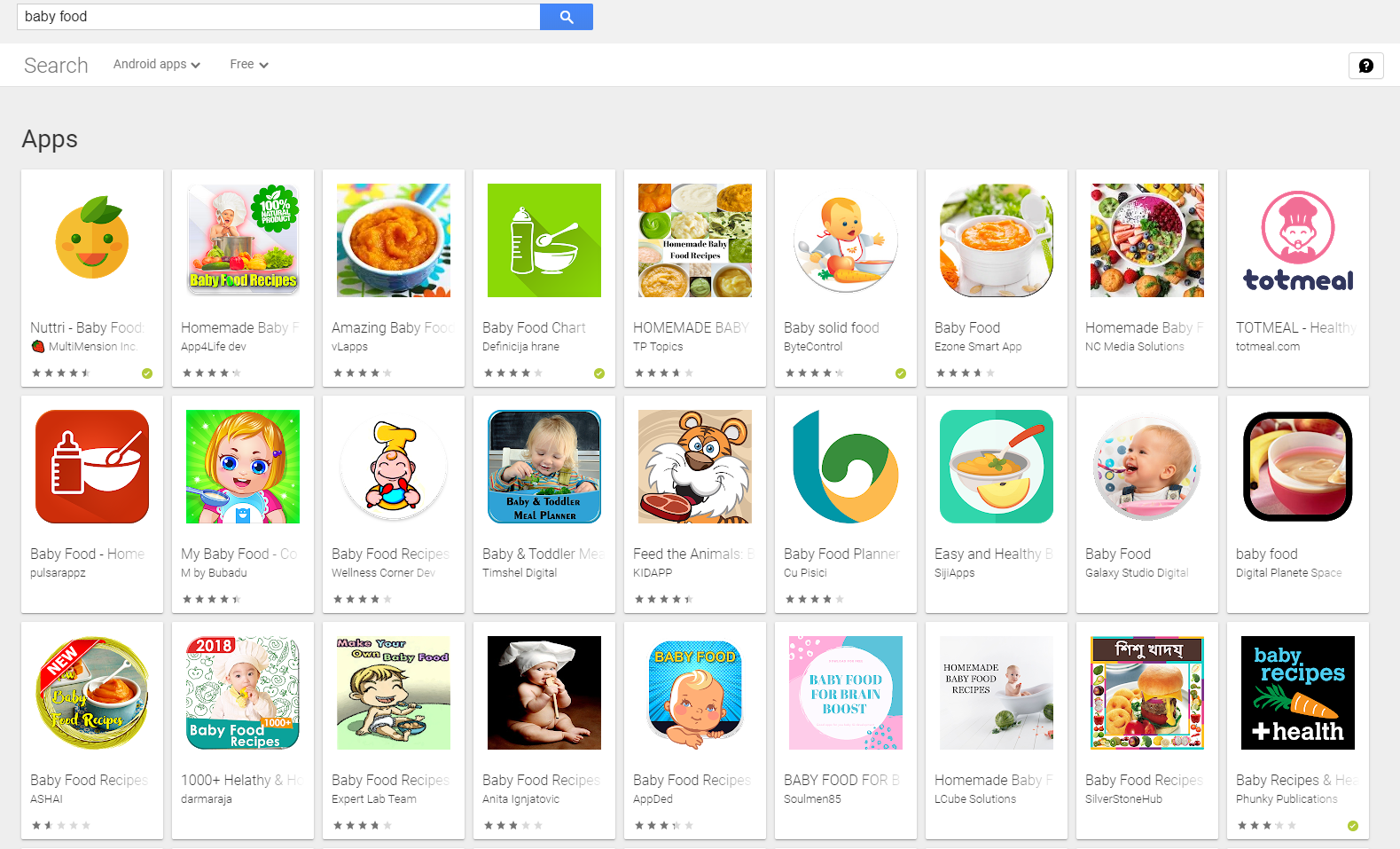


**This app looks relevant and should be checked**

**This is a game and should be excluded**

**This is farmed content and should be excluded**

# Stage 1

## Identification and selection of the applications

Applications will be identified using the following approaches:

1. Select app store – App Store or Google Play (<https://play.google.com/store>).

- For Google Play searches, do this search on the computer – the search results for apps may change if the search was done on a tablet or phone, because Google Play filters suitable apps for the device you are using.

Ensure that you log out of any Google Accounts and erase cookies from web browser history. This will avoid your previous search history influencing the search results.

- For App Store searches, this search must now be done on your own smartphone, as September 2017 iOS software updates has removed app searches on desktop through iTunes.

2. Search each of the key terms from Set 1, 2 or 3 individually (Table 1) in each search engine.

3. For App Store searches, verify your search on <https://fnd.io/> using the filter ‘Apps’ (to search only for apps) and the store ‘Australia’ (to search for software available in Australia).

Record information of each search, including:

- App store used
- Key terms
- Date of search conducted
- Free or paid app search
- Total number of results found
- Number of apps included, excluded, unsure on inclusion/exclusion^4^, and with broken links

1. After identification of relevant apps for inclusion, proceed with assessment and evaluation of apps.

^4^ Uncertain apps will be recorded and reviewed by another researcher. Discrepancies will be resolved by discussion.

# Stage 2

## Assessment of information in smartphone apps

Validated tools are used to assess the quality, readability and suitability of smartphone applications on infant feeding practices, promoting healthy infant growth and infant activity.

Evidence-based guidelines ‘Infant Feeding Guidelines’ (2012), ‘National Physical Activity Recommendations for Children 0-5 Years’ (2010) and primary literature were used to develop the criteria to assess accuracy and coverage of the content of apps.

The following needs to be assessed from the selected apps:

1. Accurate coverage and depth of information – using criteria based on the Infant Feeding Guidelines, National Physical Activity Recommendations for Children 0-5 Years and primary literature
2. App quality – using the Mobile Application Rating Scale (MARS) tool
3. Suitability of information – using the Suitability Assessment of Material (SAM)
4. Readability of information – using the Simple Measure of Gobbledygook (SMOG) and Flesch-Kincaid (F-K) tools
5. App usability – using two reviewer-designed subscales on user Accessibility and data Security as part of the MARS tool

### I. Accurate coverage and depth of information

An information guide sheet was developed to identify the accurate coverage (referred thereafter as ‘coverage’) and depth of the infant feeding and activity covered in the app.

The content of the information sheet was derived from the variables of interest included in the inclusion criteria.

Eight topics with 22 subtopics were identified using the National Health and Medical Research Council’s Infant Feeding Guidelines (2012), the Department of Health and Ageing’s National Physical Activity Recommendations for Children 0-5 Years (2010) and primary literature. These were used to determine adequate coverage of the relevant material and the scientific accuracy of this information.

A scoring system was used to measure the coverage of the content which was derived from the Health-Related Website Evaluation Form [1], a tool used to assess the accuracy of health information websites. Coverage was scored as either correct (+1), incorrect (-1), absent (0) or not applicable, to measure how *many topics* on breastfeeding, formula feeding, introduction of solids, or infant activity were accurately covered in each app.

The final coverage score was given using the ratings from the Health-Related Website Evaluation Form [1], as excellent (90% or higher), adequate (75-89%) or poor (less than 75%).

If the subtopic was scored incorrect for coverage, it was automatically scored as incorrect for depth.

A scoring system was developed to measure the depth of the content. Depth was scored as either complete (+1), partially complete (+0.5) or incomplete or incorrect (0), to *measure how extensively* *or completely* the topic of breastfeeding or formula feeding, introduction of solids or infant activity were covered in each app.

The final depth score was given as complete (100%), partial (50-99%), or low or no (less than 49%) completeness of the addressed subtopics.

### II. Quality assessment

The quality of smartphone apps was assessed using the MARS tool.

The criteria of the MARS tool includes:

- Engagement – “fun, interesting, customisable, interactive (e.g. sends alerts, messages, reminders, feedback, enables sharing), well-targeted to audience”
- Functionality – “app functioning, easy to learn, navigation, flow logic, and gestural design of app”
- Aesthetics – “graphic design, overall visual appeal, colour scheme, and stylistic consistency”
- Information – “contains high quality information (e.g. text, feedback, measures, references) from a credible source”
- Subjective individual interest [2]

The MARS tool is scored corresponding to a 5-star rating system, receiving a score of 1 (lowest) to 5 (highest). The scoring system is described in-depth in a corresponding training video [3].

A final measurement of app quality is calculated using the mean from scores from the Engagement, Functionality, Aesthetics and Information quality sections.

The subjective quality of the app is calculated as a separate rating, as a mean of the Subjective individual interest section.

### III. Suitability of information

The SAM was used to objectively assess the suitability of the information in apps [4]. The SAM tool is assessed using various areas of the application including: content, literacy level, graphics, layout, interaction with readers, learning stimulation and motivation and cultural appropriateness.

A scoring system of superior (+2), adequate (+1), not suitable (0) or not applicable, is reported based on the objective criteria of the instrument.

The F-K and SMOG reading grade is used for every app is used to determine the ‘reading grade level’ (section IV, readability of information).

A final score for appropriateness of information was calculated as being superior (70-100%), adequate (40-69%), or not suitable (0-39%).

The hypothetical target audience used in app evaluation were multiethnic Australians with an Australian Year 3 to 4 reading level, with the reading level consistent with Australian government Plain English guidelines [5].

### IV. Readability of information

Multiple readability tools may be used to establish the reading levels of the applications based on the complexity of words and sentences [6]. The two most commonly used tools include F-K [7] and SMOG [8] test, and were used in this evaluation.

A section of text from the apps were assessed using the <http://www.readabilityformulas.com/free-readability-formula-tests.php> website, and the results of the F-K and SMOG tests recorded.

The F-K test was repeated on the same section of text, with the built-in Microsoft Word 2010, 2013 or 2016 function and using the following instructions on using the readability function, <https://support.office.com/en-ie/article/test-your-document-s-readability-85b4969e-e80a-4777-8dd3-f7fc3c8b3fd2>, with the results recorded.

The SMOG and F-K tests rate sections of text on an American school grade level. The Australian government Plain English guidelines [5] recommends writing for an Australian Year 3 to 4 reading level, or the equivalent American Grade 3 to 4 reading level. In the SAM, readability measured by the F-K and SMOG is scored as superior (Grade 5 and lower reading level), adequate (Grade 6 to 8 reading level), or not suitable (Grade 9 or higher reading level).

The F-K and SMOG scores calculated from the online readability calculator and on Microsoft Word software were compared using the Spearman rank-order correlation using IBM SPSS Statistics for Windows, Version 25.0 (Armonk, NY: IBM Corp).

### V. App usability

Two scales to assess the usability quality of apps, for criteria not described in other tools, were included [9].

These scales on Data security, defined as data encryption and privacy; and Accessibility, defined as multilanguage options, one-handed functionality, and availability of help guides, were added to the MARS.

The mean of the scores from the Data security and Accessibility sections were calculated and included in a separate calculation of the MARS score, which was then referred to as the modified MARS score.

## Summary of steps

The following steps for **stage 2** are summarised as follows:

1. Assess coverage and depth of information on infant feeding, introduction to solids and/or infant activity.
2. Measure readability using the SMOG and F-K tests – these scores will be used in the SAM tool.
3. Assess the suitability of information using the SAM tool.
4. Assess quality of apps using the MARS tool, with additional Data security and Accessibility scales.

# Stage 3

## Inter-rater reliability

At least 20% of apps were randomly selected for assessment by a second reviewer.

Inter-rater reliability in the accuracy and coverage of information, MARS score, SAM score, and readability tests were calculated using a macro extension to calculate Krippendorff’s alpha [10] on IBM SPSS Statistics for Windows, Version 25.0 (Armonk, NY: IBM Corp).

# References

1. Health on the Net Foundation. *Health-Related Web Site Evaluation Form*. [cited 2019 21 January 2019]; Available from: <https://www.carlbring.se/form/itform_eng.pdf>.

2. Stoyanov, S.R., et al., *Mobile App Rating Scale: A New Tool for Assessing the Quality of Health Mobile Apps.* JMIR mHealth and uHealth, 2015. **3**(1): p. e27.

3. Stoyanov, S.R., *MARS training video*. 2016, YouTube.

4. Doak, C., L. Doak, and J. Root. *Suitability Assessment of Materials for evaluation of health-related information for adults*. 2008 [cited 2019 21 January 2019]; Available from: <http://aspiruslibrary.org/literacy/sam.pdf>.

5. GOV.AU. *Writing style*. 2019 [cited 2019 21 January 2019]; Available from: <https://guides.service.gov.au/content-guide/writing-style/>.

6. Eysenbach, G., et al., *Empirical studies assessing the quality of health information for consumers on the world wide web: a systematic review.* JAMA, 2002. **287**(20): p. 2691-700.

7. Kincaid, P.J., et al. *Derivation Of New Readability Formulas (Automated Readability Index, Fog Count And Flesch Reading Ease Formula) For Navy Enlisted Personnel*. 1975 1 January 1975 Research Branch Report 8-75]; Available from: <https://stars.library.ucf.edu/cgi/viewcontent.cgi?article=1055&context=istlibrary>.

8. McLaughlin, H.G. *SMOG Grading - a New Readability Formula*. Journal of Reading, 1969. **12**, 639-646.

9. Taki, S., et al., *Infant Feeding Websites and Apps: A Systematic Assessment of Quality and Content.* Interactive Journal of Medical Research, 2015. **4**(3): p. e18.

10. Hayes, A.F. *My Macros and Code for SPSS and SAS*. 2007 [cited 2019 9 September]; Available from: <http://afhayes.com/spss-sas-and-mplus-macros-and-code.html>.
