# Supplementary material for: Content and Quality of Infant Feeding Smartphone Apps: Five-Year Update on a Systematic Search and Evaluation
Source: JMIR Mhealth Uhealth. 2020 May 27;8(5):e17300. doi: 10.2196/17300 (PMC7287747; doi:10.2196/17300)
Supplement: Multimedia Appendix 3 [file mhealth_v8i5e17300_app3.pdf]

Mobile App Rating Scale

Record ID

____

Name of smartphone app

____

Rating of this version of smartphone app

____

Rating of all versions of smartphone app

____

Developer

____

Which version of the app is this?

____

When was this app last updated?

____

What is the cost of the basic version of the app?

____

What is the cost of the upgraded version of the app?

____
(This can be 'in-app purchases' for an app that is free to download; or a separate 'paid/premium/pro' version of the app that must be paid for at download.)

What platform are you using this app on?

◯ iPhone
◯ Android

Please provide a brief description of this app - see its app store listing About section as a guide.

____

Which app store search did you find this app in?

☐ Infant feeding
☐ Introducing solids
☐ Infant activity

What theoretical background or strategies does this app use?

☐ Assessment
☐ Feedback
☐ Information/education
☐ Monitoring/tracking
☐ Goal setting
☐ Advice/tips/strategies/skills training

Are there organisations or groups affiliated with this app?	☐ Unknown/do not know<br>☐ Commercial<br>☐ Government<br>☐ Non-government organisations<br>☐ University<br>☐ Does not have any affiliations
What are the technical aspects of this app?	☐ Allows sharing on social media (Facebook, Twitter, etc.)<br>☐ Has an app community<br>☐ Allows password protection<br>☐ Requires login<br>☐ Sends reminders<br>☐ Needs internet access to function
If there are other technical aspects of this app not covered, describe them here.	__________________________________

The Mobile Application Rating Scale (MARS) assesses app quality on four dimensions. All items are rated on a 5-point scale from "1. Inadequate" to "5. Excellent". Select the number that most accurately represents the quality of the app component you are rating. Please use the descriptors provided for each response category.

The MARS is used to evaluate apps.

Note - you may want to complete:
- the "Website and app content based on 2012 Infant Feeding Guidelines"
- the "Suitability Assessment of Material"
forms first to assess the app - this can help guide your Section D response.

SECTION A: Engagement - fun, interesting, customisable, interactive (e.g. sends alerts, messages, reminders, feedback, enables sharing), well-targeted to audience

1. Entertainment: Is the app fun/entertaining to use? Does it use any strategies to increase engagement through entertainment (e.g. through gamification)?	○ 1. Dull, not fun or entertaining at all<br>○ 2. Mostly boring<br>○ 3. OK, fun enough to entertain user for a brief time (< 5 minutes)<br>○ 4. Moderately fun and entertaining, would entertain user for some time (5-10 minutes total)<br>○ 5. Highly entertaining and fun, would stimulate repeat use
2. Interest: Is the app interesting to use? Does it use any strategies to increase engagement by presenting its content in an interesting way?	○ 1. Not interesting at all<br>○ 2. Mostly uninteresting<br>○ 3. OK, neither interesting nor uninteresting; would engage user for a brief time (< 5 minutes)<br>○ 4. Moderately interesting; would engage user for some time (5-10 minutes total)<br>○ 5. Very interesting, would engage user in repeat use

3. Customisation: Does it provide/retain all necessary settings/preferences for apps features (e.g. sound, content, notifications, etc.)?	◯ 1. Does not allow any customisation or requires setting to be input every time<br>◯ 2. Allows insufficient customisation limiting functions<br>◯ 3. Allows basic customisation to function adequately<br>◯ 4. Allows numerous options for customisation<br>◯ 5. Allows complete tailoring to the individual's characteristics/preferences, retains all settings
4. Interactivity: Does it allow user input, provide feedback, contain prompts (reminders, sharing options to social media/through email or Bluetooth, notifications, etc.)? Note: these functions need to be customisable and not overwhelming in order to be perfect.	◯ 1. No interactive features and/or no response to user interaction<br>◯ 2. Insufficient interactivity, or feedback, or user input options, limiting functions<br>◯ 3. Basic interactive features to function adequately<br>◯ 4. Offers a variety of interactive features/feedback/user input options<br>◯ 5. Very high level of responsiveness through interactive features/feedback/user input options
5. Target group: Is the app content (visual information, language, design) appropriate for your target audience?	◯ 1. Completely inappropriate/unclear/confusing<br>◯ 2. Mostly inappropriate/unclear/confusing<br>◯ 3. Acceptable but not targeted. May be inappropriate/unclear/confusing<br>◯ 4. Well-targeted, with negligible issues<br>◯ 5. Perfectly targeted, no issues found
Admin only: Mean Engagement score.	________________________________

SECTION B: Functionality - app functioning, easy to learn, navigation, flow logic, and gestural design of app

6. Performance: How accurately/fast do the app features (functions) and components (buttons/menus) work?	◯ 1. App is broken; no/insufficient/inaccurate response (e.g. crashes/bugs/broken features, etc.)<br>◯ 2. Some functions work, but lagging or contains major technical problems<br>◯ 3. App works overall. Some technical problems need fixing/slow at times<br>◯ 4. Mostly functional with minor/negligible problems<br>◯ 5. Perfect/timely response; no technical bugs found/contains a 'loading time left' indicator
7. Ease of use: How easy is it to learn how to use the app; how clear are the menu labels/icons and instructions?<br><br>E.g. are the functions of the app intuitive and do they operate as expected?	◯ 1. No/limited instructions; menu labels/icons are confusing; complicated<br>◯ 2. Useable after a lot of time/effort<br>◯ 3. Useable after some time/effort<br>◯ 4. Easy to learn how to use the app (or has clear instructions)<br>◯ 5. Able to use app immediately; intuitive; simple

8. Navigation: Is moving between screens logical/accurate/appropriate/uninterrupted; are all necessary screen links present?<br><br>E.g. navigation between screens should be able to be reached in as few actions as possible - from the main page of the subsection (e.g. a new record section, or result section), it should not take more than three navigational actions to reach a page.<br><br>Navigation should also be logical, intuitive and clearly indicated, with signs obvious and not obscured.	◯ 1. Different sections within the app seem logically disconnected and random/confusing/navigation is difficult<br>◯ 2. Usable after a lot of time/effort<br>◯ 3. Usable after some time/effort<br>◯ 4. Easy to use or missing a negligible link<br>◯ 5. Perfectly logical, easy, clear and intuitive screen flow throughout, or offers shortcuts
9. Gestural design: Are interactions (taps/swipes/pinches/scrolls) consistent and intuitive across all components/screens?<br><br>E.g. interactive items should be stylistically indicated as being able to be tapped, swiped, pinched or scrolled.	◯ 1. Completely inconsistent/confusing<br>◯ 2. Often inconsistent/confusing<br>◯ 3. OK with some inconsistencies/confusing elements<br>◯ 4. Mostly consistent/intuitive with negligible problems<br>◯ 5. Perfectly consistent and intuitive
Admin only: Mean Functionality score.	__________________________

SECTION C: Aesthetics - graphic design, overall visual appeal, colour scheme, and stylistic consistency

10. Layout: Is arrangement and size of buttons/icons/menus/content on the screen appropriate or zoomable if needed?	◯ 1. Very bad design, cluttered, some options impossible to select/locate/see/read device display not optimised<br>◯ 2. Bad design, random, unclear, some options difficult to select/locate/see/read<br>◯ 3. Satisfactory, few problems with selecting/locating/seeing/reading items or with minor screen size problems<br>◯ 4. Mostly clear, able to select/locate/see/read items<br>◯ 5. Professional, simple, clear, orderly, logically organised, device display optimised. Every design component has a purpose
11. Graphics: How high is the quality/resolution of graphics used for buttons/icons/menus/content?	◯ 1. Graphics appear amateur, very poor visual design - disproportionate, completely stylistically inconsistent<br>◯ 2. Low quality/low resolution graphics; low quality visual design - disproportionate, stylistically inconsistent<br>◯ 3. Moderate quality graphics and visual design (generally consistent in style)<br>◯ 4. High quality/resolution graphics and visual design - mostly proportionate, stylistically consistent<br>◯ 5. Very high quality/resolution graphics and visual design - proportionate, stylistically consistent throughout

12. Visual appeal: How good does the app look?<br><br>Consider:<br>- consistent colour scheme<br>- visually accessible colour choices (easy to read with normal or corrected vision)<br>- legible font sizes, consistent font themes (1-2 fonts used logically, not different fonts used inconsistently)	◯ 1. No visual appeal, unpleasant to look at, poorly designed, clashing/mismatched colours<br>◯ 2. Little visual appeal - poorly designed, bad use of colour, visually boring<br>◯ 3. Some visual appeal - average, neither pleasant, nor unpleasant<br>◯ 4. High level of visual appeal - seamless graphics - consistent and professionally designed<br>◯ 5. As above + very attractive, memorable, stands out; use of colour enhances app features/menus
Admin only: Mean Aesthetics score.	________________________

SECTION D: Information - Contains high quality information (e.g. text, feedback, measures, references) from a credible source. Select N/A if the app component is irrelevant.

13. Accuracy of app description (in app store): Does app contain what is described in its About section?	◯ 1. Misleading. App does not contain the described components/functions. Or has no description<br>◯ 2. Inaccurate. App contains very few of the described components/functions<br>◯ 3. OK. App contains some of the described components/functions<br>◯ 4. Accurate. App contains most of the described components/functions<br>◯ 5. Highly accurate description of the app components/functions
14. Goals: Does app have specific, measurable and achievable goals (specified in app store description or within the app itself)?	◯ N/A. Description does not list goals, or app goals are irrelevant to research goal (e.g. using a game for educational purposes)<br>◯ 1. App has no chance of achieving its stated goals<br>◯ 2. Description lists some goals, but app has very little chance of achieving them<br>◯ 3. OK. App has clear goals, which may be achievable.<br>◯ 4. App has clearly specified goals, which are measurable and achievable<br>◯ 5. App has specific and measurable goals, which are highly likely to be achieved
15. Quality of information: Is app content correct, well written, and relevant to the goal/topic of the app?<br><br>Note: refer to your overall score for this app on the "Suitability Assessment of Material" form.<br><br>Suggestion:<br>For "70-100%, superior material" ratings, score 4 or 5 here.<br>For "40-69%, adequate material" ratings, score 3 or 4 here.<br>For "0-39%, not suitable material" ratings, score 1 or 2 here.	◯ N/A. There is no information within the app<br>◯ 1. Irrelevant/inappropriate/incoherent/incorrect<br>◯ 2. Poor. Barely relevant/appropriate/coherent/may be incorrect<br>◯ 3. Moderately relevant/appropriate/coherent/and appears correct<br>◯ 4. Relevant/appropriate/coherent/correct<br>◯ 5. Highly relevant, appropriate, coherent, and correct

16. Quantity of information: Is the extent coverage within the scope of the app; and comprehensive but concise?

Note: refer to your overall score for this app on the "Website and app content based on 2012 Infant Feeding Guidelines" form.

Suggestion:
For "≥90%, excellent" ratings, score 5 here.
For "75-89%, adequate" ratings, score 3 or 4 here.
For "≤74%, poor" ratings, score 1 or 2 here.

- ◯ N/A. There is no information within the app
- ◯ 1. Minimal or overwhelming
- ◯ 2. Insufficient or possibly overwhelming
- ◯ 3. OK but not comprehensive or concise
- ◯ 4. Offers a broad range of information, has some gaps or unnecessary detail; or has no links to more information and resources
- ◯ 5. Comprehensive and concise; contains links to more information and resources

17. Visual information: Is visual explanation of concepts - through charts/graphs/images/videos, etc. - clear, logical, correct?

- ◯ N/A. There is no visual information within the app (e.g. it only contains audio, or text)
- ◯ 1. Completely unclear/confusing/wrong or necessary but missing
- ◯ 2. Mostly unclear/confusing/wrong
- ◯ 3. OK but often unclear/confusing/wrong
- ◯ 4. Mostly clear/logical/correct with negligible issues
- ◯ 5. Perfectly clear/logical/correct

18. Credibility: Does the app come from a legitimate source (specified in app store description or within the app itself)?

Note: legitimacy of the source will include professional input - e.g. from a clinician, nutritionist, dietitian, nurse, midwife, paediatrician, lactation consultant, physiotherapist, occupational therapist or physician

- ◯ 1. Source identified but legitimacy/trustworthiness of source is questionable (e.g. commercial business with vested interest)
- ◯ 2. Appears to come from a legitimate source, but it cannot be verified (e.g. has no webpage)
- ◯ 3. Developed by small NGO/institution (hospital/centre, etc.) /specialised commercial business, funding body
- ◯ 4. Developed by government, university or as above but larger in scale
- ◯ 5. Developed using nationally competitive government or research funding (e.g. Australian Research Council, NHMRC)

An example of searching specifically for smartphone apps or mHealth topics is attached.

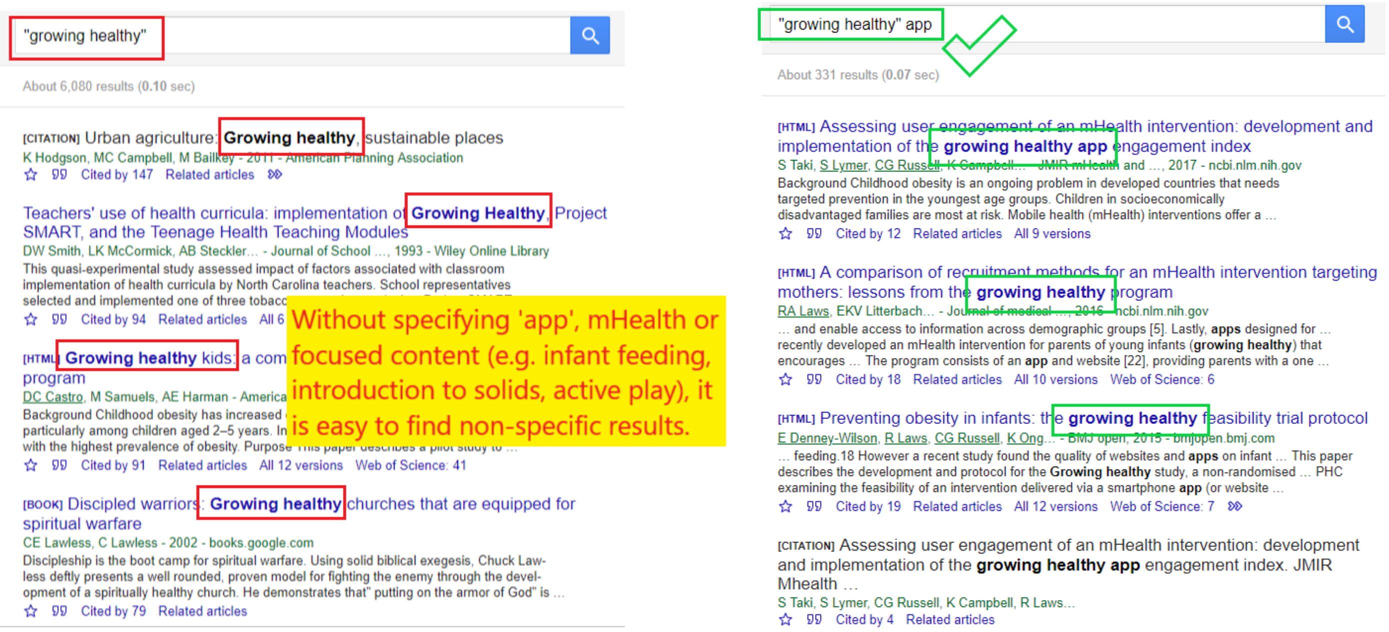

19. Evidence base: Has the app been trialled/tested; must be verified by evidence (in published scientific literature)?<br><br>Note: to check if this app has been published in scientific literature, search for the name of the app in Google Scholar.	◯ N/A. The app has not been trialled/tested<br>◯ 1. The evidence suggests the app does not work<br>◯ 2. App has been trialled (e.g., acceptability, usability, satisfaction ratings) and has partially positive outcomes in studies that are not randomised controlled trials (RCTs), or there is little or no contradictory evidence.<br>◯ 3. App has been trialled (e.g., acceptability, usability, satisfaction ratings) and has positive outcomes in studies that are not RCTs, and there is no contradictory evidence.<br>◯ 4. App has been trialled and outcome tested in 1-2 RCTs indicating positive results<br>◯ 5. App has been trialled and outcome tested in >3 high quality RCTs indicating positive results
Admin only: Sum of Information scores (will not include N/A scores).	__________________________
Admin only: Possible points for Section D (does not include N/A scores).	__________________________
Admin only: Mean Information score.	__________________________

SECTION E: App subjective quality

20. Would you recommend this app to people who might benefit from it?	◯ 1. Not at all - I would not recommend this app to anyone<br>◯ 2. There are very few people I would recommend this app to<br>◯ 3. Maybe - There are several people whom I would recommend it to<br>◯ 4. There are many people I would recommend this app to<br>◯ 5. Definitely - I would recommend this app to everyone
21. How many times do you think you would use this app in the next 12 months if it was relevant to you?	◯ 1. None<br>◯ 2. 1-2<br>◯ 3. 3-10<br>◯ 4. 10-50<br>◯ 5. >50
22. Would you pay for this app?	◯ 1. No<br>◯ 3. Maybe<br>◯ 5. Yes
23. What is your overall star rating of the app?	◯ * One of the worst apps I've used<br>◯ **<br>◯ *** Average<br>◯ ****<br>◯ ***** One of the best apps I've used

REVIEWER-DESIGNED SUBSCALE: Accessibility

24. Is the app available in other languages?
- ◯ No
- ◯ Yes

25. Can data be easily inputted into this app, reducing or avoiding the need for user to use both hands?
- ◯ No
- ◯ Yes
- ◯ Not applicable

26. Does the application provide a help or user guide?

Check your response to Q7.
- ◯ No
- ◯ Yes
- ◯ Not applicable

27. Does the application provide a way of contacting the developers for support?

Note: you should be able to do this as part of the app, without going back to the App Store or Google Play listing.
- ◯ No or not found
- ◯ Yes

Admin only: Sum of Accessibility (subscale) score. ________________________

Admin only: Possible points for Accessibility subscale (does not include N/A scores). ________________________

Admin only: Mean Accessibility (subscale) score. ________________________

REVIEWER-DESIGNED SUBSCALE: Security

28. User data should be kept private and safe - is the data inputted into the app encrypted in the event of loss or system malfunction?
- ◯ No or not found
- ◯ Yes
- ◯ Not applicable

29. Can information be backed up or restored, in case of device malfunction, data loss or deletion of app?
- ◯ No or not found
- ◯ Yes
- ◯ Not applicable

30. Does the app developer/publisher clearly state the privacy policy regarding how confidential, private or semi-private information will be treated?
- ◯ No or not found
- ◯ Yes
- ◯ Not applicable

Admin only: Sum of Security (subscale) score. ________________________

Admin only: Possible points for Security subscale (does not include N/A scores). ________________________

Admin only: Mean Security (subscale) score. ________________________

SCORING

Admin only: App subjective quality mean score

____

Admin only: modified MARS app quality mean score

Mean of sections A, B, C, D, Accessibility subscale, Security subscale

____

Admin only: MARS app quality mean score

Mean of sections A, B, C, D only

____

Is the MARS app quality score (mean of sections A to D) the same as the modified MARS app quality score (mean of sections A to D, Accessibility subscale, Security subscale)?

- ◯ No
- ◯ Yes

App content based on 2012 Infant Feeding Guidelines

Record ID ______________________

Topics were identified using the National Health and Medical Research Council's Infant Feeding Guidelines (2012), the Department of Health and Ageing's National Physical Activity Recommendations for Children 0-5 Years (2010) and primary literature. These were used to determine adequate coverage of the relevant material and the scientific accuracy of this information on apps.

Please select answers based on the focus of the smartphone app (i.e. infant feeding, introducing solids or infant activity) and select N/A for the other sections that are not applicable.

Name of smartphone app ______________________

Which app store search did you find this app in?
- ☐ Infant feeding
- ☐ Introducing solids
- ☐ Infant activity

What platform are you using this app on?
- ○ iPhone
- ○ Android

Is the revision/last update date for this app recent enough to account for changes in the field?

i.e. is the revision/last update date after the publication of the NHMRC Infant Feeding Guidelines in 2012 or the National Physical Activity Recommendations for Children 0-5 years olds in 2010?
- ○ No or no last update date given
- ○ Yes

COVERAGE of infant feeding content in apps

	Correct advice (+1)	Incorrect advice (-1)	Not addressed (0)	N/A - not the focus of this app
Encouraging and supporting breastfeeding: Breastfeeding as the physiological norm	○	○	○	○
Encouraging and supporting breastfeeding: Protection and promotion of breastfeeding	○	○	○	○
Initiating breastfeeding: Breastfeeding education for parents	○	○	○	○
Initiating breastfeeding: Physiology of breast milk and breastfeeding	○	○	○	○
Initiating breastfeeding: The first breastfeed	○	○	○	○

Establishing and maintaining breastfeeding: Difficulty establishing breastfeeding	○	○	○	○
Establishing and maintaining breastfeeding: Factors affecting establishment of breastfeeding	○	○	○	○
Establishing and maintaining breastfeeding: Monitoring an infant's progress	○	○	○	○
Establishing and maintaining breastfeeding: Maternal nutrition	○	○	○	○
Breastfeeding, common problems and their management: Maternal factors affecting breastfeeding	○	○	○	○
Breastfeeding, common problems and their management: Infant factors affecting breastfeeding	○	○	○	○
Expressing and storing breast milk: Expressing breast milk	○	○	○	○
Expressing and storing breast milk: Feeding with expressed breast milk	○	○	○	○
Expressing and storing breast milk: Storage of expressed breast milk	○	○	○	○
Breastfeeding in specific situations: Tobacco, alcohol and other drugs	○	○	○	○
Infant formula: Preparing infant formula	○	○	○	○
Infant formula: Using infant formula	○	○	○	○
Infant formula: Special infant formula	○	○	○	○

DEPTH of infant feeding content in apps.

If not applicable, this section will stay blank.

For the above infant feeding topics that were available in the app, score this information as being 'complete', 'partially complete' or 'incomplete or incorrect'.

	Partially complete (+0.5)	Complete (+1)	Incomplete or incorrect information (0)
Encouraging and supporting breastfeeding: Breastfeeding as the physiological norm	○	○	○
Encouraging and supporting breastfeeding: Protection and promotion of breastfeeding	○	○	○
Initiating breastfeeding: Breastfeeding education for parents	○	○	○
Initiating breastfeeding: Physiology of breast milk and breastfeeding	○	○	○
Initiating breastfeeding: The first breastfeed	○	○	○
Establishing and maintaining breastfeeding: Difficulty establishing breastfeeding	○	○	○
Establishing and maintaining breastfeeding: Factors affecting establishment of breastfeeding	○	○	○
Establishing and maintaining breastfeeding: Monitoring an infant's progress	○	○	○
Establishing and maintaining breastfeeding: Maternal nutrition	○	○	○
Breastfeeding, common problems and their management: Maternal factors affecting breastfeeding	○	○	○
Breastfeeding, common problems and their management: Infant factors affecting breastfeeding	○	○	○
Expressing and storing breast milk: Expressing breast milk	○	○	○

Expressing and storing breast milk: Feeding with expressed breast milk	○	○	○
Expressing and storing breast milk: Storage of expressed breast milk	○	○	○
Breastfeeding in special situations: Tobacco, alcohol and other drugs	○	○	○
Infant formula: Preparing infant formula	○	○	○
Infant formula: Using infant formula	○	○	○
Infant formula: Special infant formula	○	○	○

ADMIN ONLY: Coverage of infant feeding content ________________________

ADMIN ONLY: Depth of infant feeding content ________________________

ADMIN ONLY: Total of number of points possible on infant feeding content. ________________________

COVERAGE of introducing solids content in apps

	Correct advice (+1)	Incorrect advice (-1)	Not addressed (0)	N/A - not the focus of this app
Introducing solids: When should solid foods be introduced?	○	○	○	○
Introducing solids: What foods should be introduced	○	○	○	○
Introducing solids: Foods and beverages most suitable for infants, or that should be used in care	○	○	○	○
Introducing solids: Healthy foods in the first 12 months (continued exposure and opportunity to sample a wide variety of healthy foods)	○	○	○	○

DEPTH of introducing solids content in apps.

If not applicable, this section will stay blank.

For the above introducing solids topics that were available in the app, score this information as being 'complete', 'partially complete' or 'incomplete or incorrect'.

	Partially complete (+0.5)	Complete (+1)	Incomplete or incorrect information (0)
Introducing solids: When should solid foods be introduced?	○	○	○
Introducing solids: What solid foods should be introduced	○	○	○
Introducing solids: Foods and beverages most suitable for infants, or that should be used in care	○	○	○
Introducing solids: Healthy foods in the first 12 months (continued exposure and opportunity to sample a wide variety of healthy foods)	○	○	○

ADMIN ONLY: Coverage of introducing solids content ________________________________

ADMIN ONLY: Depth of introducing solids content ________________________________

ADMIN ONLY: Total of number of points possible on infant feeding content. ________________________________

COVERAGE of infant activity content in apps

	Correct advice (+1)	Incorrect advice (-1)	Not addressed (0)	N/A - not the focus of this app
Infant activity: Encouraging physical activity for infants from birth for healthy development	○	○	○	○

Infant activity: Advice on types of infant physical activity/movements for development including; reaching and grasping, pulling and pushing, moving their head, body and limbs during daily routines, and supervised floor play, including tummy time	○	○	○	○

DEPTH of infant activity content in apps.

If not applicable, this section will stay blank.

For the above infant activity topics that were available in the app, score this information as being 'complete', 'partially complete' or 'incomplete or incorrect'.

	Partially complete (+0.5)	Complete (+1)	Incomplete or incorrect information (0)
Infant activity: Encouraging physical activity for infants from birth for healthy development	○	○	○
Infant activity: Advice on types of infant physical activity/movements for development including; reaching and grasping, pulling and pushing, moving their head, body and limbs during daily routines, and supervised floor play, including tummy time	○	○	○

ADMIN ONLY: Coverage of infant activity content ______________________

ADMIN ONLY: Depth of infant activity content ______________________

Admin only: Total of number of points possible for infant activity content. ______________________

SCORING

ADMIN ONLY: Total of all coverage scores, including 1 point for currency	__________________________
ADMIN ONLY: Total of all depth scores, including 1 point for currency	__________________________
ADMIN ONLY: Total of all possible points, including 1 point for currency	__________________________
ADMIN ONLY: Overall coverage rating score (%)	__________________________
Please select the overall coverage rating, corresponding to the above.	◯ ≥90%, excellent<br>◯ 75-89%, adequate<br>◯ ≤74%, poor
ADMIN ONLY: Overall depth rating score (%)	__________________________
Please select the overall depth rating, corresponding to the above.	◯ 100%, complete coverage<br>◯ 50-99%, partial coverage<br>◯ ≤49%, low or no coverage

Suitability Assessment of Material

Record ID ______________________________

Note: make sure you do the Simple Measure of Gobbledygook test and the Flesch-Kincaid readability tests first - this is a component of the Suitability Assessment of Material (SAM) form.

Instructions:

1. Read through the SAM factor list and the evaluation criteria on the score sheet. It is best to have a copy of the SAM beside you as you evaluate using this form - it has been abbreviated for us in this form.

A copy of the SAM is attached to this form.

2. Read the material (the app) you want to evaluate and determine its purpose(s) and key points.

3. For short documents (for example a pamphlet or single page of text), evaluate the entire piece.

For longer documents (for example a booklet), select samples of key sections to evaluate, or three pages that cover topics central to the purpose of the booklet.

For documents longer than 50 pages, increase the sample size to six pages.

4. Evaluate and score each of the 22 SAM factors using the evaluation criteria provided, and circle the appropriate score on the score sheet. The scoring system provides:
- 2 points per factor for superior rating
- 1 point per factor for adequate rating
- 0 points per factor for not suitable rating.

As you evaluate each factor, you are likely to find wide variation in different parts of your material. For any one factor, some parts may rate high (superior), while other parts may rate low (unsuitable). Resolve this by giving most weight to the part of your material that includes the key points that you identified in step 2 above.

[Attachment: "SAM.pdf"]

Name of smartphone app ______________________________

Which app store search did you find this app in?
- ☐ Infant feeding
- ☐ Introducing solids
- ☐ Infant activity

What platform are you using this app on?
- ◯ iPhone
- ◯ Android

SECTION 1 - Content

	0. Not suitable	+1. Adequate	+2. Superior	Not applicable
Purpose is evident.	◯	◯	◯	◯
Content about behaviours.	◯	◯	◯	◯
Scope is limited to essential information.	◯	◯	◯	◯

Summary and review.	○	○	○	○

Admin only: SECTION 1 score

____

SECTION 2 - Literacy Demand

	0. Not suitable	+1. Adequate	+2. Superior	Not applicable
Reading grade level.	○	○	○	○
Writing style, active voice.	○	○	○	○
Vocabulary uses common words.	○	○	○	○
Context is given first.	○	○	○	○
Headers or topic captions.	○	○	○	○

Admin only: SECTION 2 score

____

SECTION 3 - Graphics

	0. Not suitable	+1. Adequate	+2. Superior	Not applicable
Cover graphic shows purpose.	○	○	○	○
Type of illustrations.	○	○	○	○
Relevance of illustrations.	○	○	○	○
Lists, tables, graphs and charts explained.	○	○	○	○
Captions used for graphics.	○	○	○	○

Admin only: SECTION 3 score

____

SECTION 4 - Layout and typography

	0. Not suitable	+1. Adequate	+2. Superior	Not applicable
Layout factors.	○	○	○	○
Typography.	○	○	○	○
Subheadings used.	○	○	○	○

Admin only: SECTION 4 score

____

SECTION 5 - Learning, stimulation, motivation

	0. Not suitable	+1. Adequate	+2. Superior	Not applicable
Interaction with readers used.	◯	◯	◯	◯
Behaviours are modelled and specific.	◯	◯	◯	◯
Motivation with self-efficable tasks and behaviours.	◯	◯	◯	◯

Admin only: SECTION 5 score

____

SECTION 6 - Cultural appropriateness

	0. Not suitable	+1. Adequate	+2. Superior	Not applicable
Cultural match.	◯	◯	◯	◯
Cultural image and examples.	◯	◯	◯	◯

Admin only: SECTION 6 score

____

SCORING

Admin only: Total of number of points possible (44 minus [2 x each N/A response])

____

Admin only: Total SAM score (sum of all scored items)

____

Admin only: Calculated overall rating score (%)

____

Please select the overall rating, corresponding to the above overall score.

◯ 70-100%, superior material
◯ 40-69%, adequate material
◯ 0-39%, not suitable material

Readability tests

Record ID ______________________

The Flesch-Kincaid (F-K) and Simple Measure of Gobbledygook (SMOG) tools will be used to measure readability.

This measure of readability will be used when using the Suitability Assessment of Material.

NOTE: make sure you use the same block of writing in the F-K and SMOG tests.

Name of smartphone app ______________________

Which app store search did you find this app in?
- ☐ Infant feeding
- ☐ Introducing solids
- ☐ Infant activity

Which app store did you find this app in?
- ◯ iPhone
- ◯ Android

Part 1: Select a paragraph of text you will analyse.

Part 2: Calculate the SMOG and F-K grade using this website:
http://www.readabilityformulas.com/free-readability-formula-tests.php

Part 3: Calculate the F-K grade using Microsoft Word. You will need Microsoft Word 2007 or Microsoft Word 2010.

Find instructions here:
https://support.office.com/en-us/article/test-your-document-s-readability-85b4969e-e80a-4777-8dd3-f7fc3c8b3fd2

An example of where the F-K test in Microsoft Word will appear is attached.

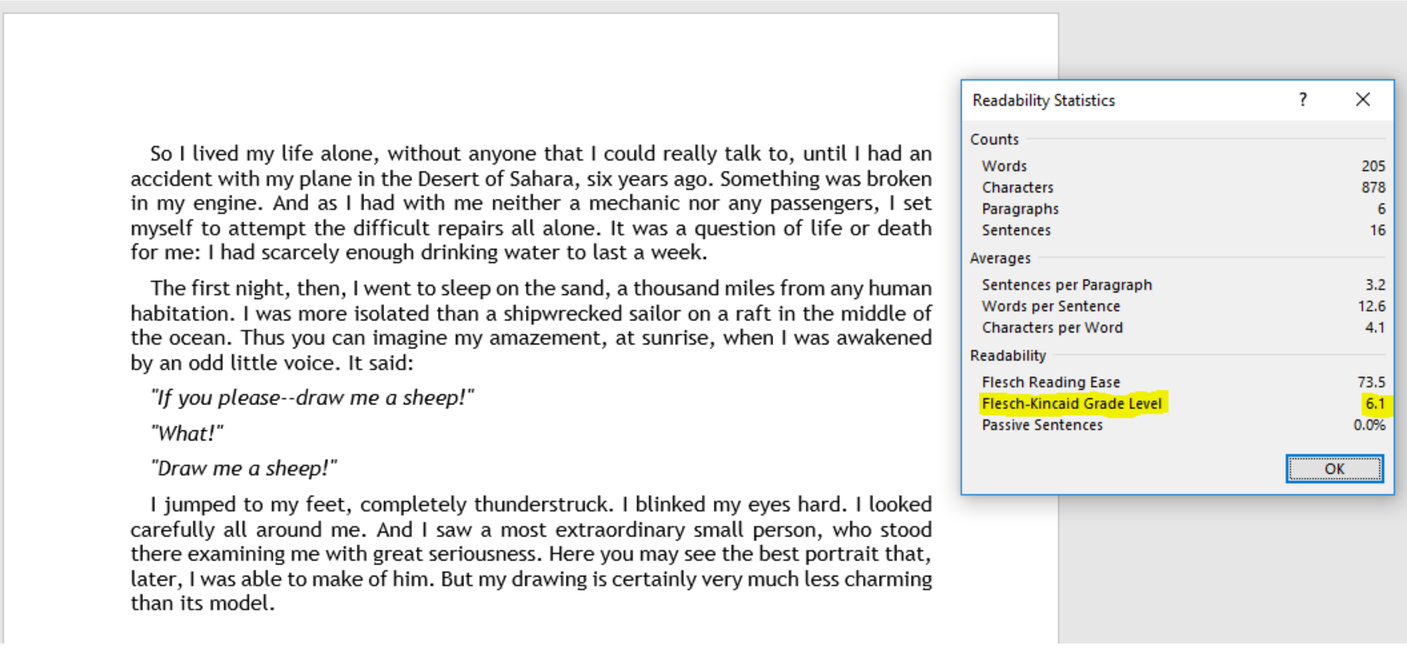

So I lived my life alone, without anyone that I could really talk to, until I had an accident with my plane in the Desert of Sahara, six years ago. Something was broken in my engine. And as I had with me neither a mechanic nor any passengers, I set myself to attempt the difficult repairs all alone. It was a question of life or death for me: I had scarcely enough drinking water to last a week.

The first night, then, I went to sleep on the sand, a thousand miles from any human habitation. I was more isolated than a shipwrecked sailor on a raft in the middle of the ocean. Thus you can imagine my amazement, at sunrise, when I was awakened by an odd little voice. It said:

"If you please--draw me a sheep!"

"What!"

"Draw me a sheep!"

I jumped to my feet, completely thunderstruck. I blinked my eyes hard. I looked carefully all around me. And I saw a most extraordinary small person, who stood there examining me with great seriousness. Here you may see the best portrait that, later, I was able to make of him. But my drawing is certainly very much less charming than its model.

Readability Statistics ? ✕

**Counts**	
Words	205
Characters	878
Paragraphs	6
Sentences	16
**Averages**	
Sentences per Paragraph	3.2
Words per Sentence	12.6
Characters per Word	4.1
**Readability**	
Flesch Reading Ease	73.5
Flesch-Kincaid Grade Level	6.1
Passive Sentences	0.0%

OK

Indicate what paragraph of text you will be analysing here. ______________________

Using the website, what is the SMOG reading grade of the text in the app you analysed?	◯ 5th grade or under<br>◯ 6th grade<br>◯ 7th grade<br>◯ 8th grade<br>◯ 9th grade<br>◯ 10th grade or over
Using the website, what is the F-K reading grade of the text in the app you analysed?	◯ 5th grade or under<br>◯ 6th grade<br>◯ 7th grade<br>◯ 8th grade<br>◯ 9th grade<br>◯ 10th grade or over
Using Microsoft Word, what is the F-K reading grade of the text in the app you analysed?	◯ 5th grade or under<br>◯ 6th grade<br>◯ 7th grade<br>◯ 8th grade<br>◯ 9th grade<br>◯ 10th grade or over
