# Supplementary material for: Content and Quality of Infant Feeding Smartphone Apps: Five-Year Update on a Systematic Search and Evaluation
Source: JMIR Mhealth Uhealth. 2020 May 27;8(5):e17300. doi: 10.2196/17300 (PMC7287747; doi:10.2196/17300)
Supplement: Multimedia Appendix 4 [file mhealth_v8i5e17300_app4.docx]

Supplementary Table 1. Inter-rater reliability of infant feeding and activity apps scored by different pairs of reviewers.

|  | | **Krippendorff’s alpha (95% CI)** |
| --- | --- | --- |
|  | |  |
| **Content** | |  |
|  | Coverage of all subtopics | .46 (-.07 - 1.00) |
|  | Depth of information of all subtopics | .55 (.10 - 1.00) |
|  | Coverage of infant feeding subtopic | .63 (-.11 - 1.00) |
|  | Depth of information of infant feeding subtopic | .71 (.13 - 1.00) |
|  | Coverage of introduction to solids subtopic | .62 (-.14 - 1.00) |
|  | Depth of information of introduction to solids subtopic | .18 (-.64 - .72) |
|  | Coverage of infant activity subtopic | .72 (.18 - 1.00) |
|  | Depth of information of infant activity subtopic | .80 (.40 - 1.00) |
| **MARS score** | |  |
|  | Subjective | .49 (-.11 - .86) |
|  | Objective | .86 (.72 - .96) |
|  | Modified | .77 (.48 - .96) |
| **Suitability Assessment of Material** | |  |
|  | Suitability Assessment of Material score | .03 (-.55 - .61) |
| **Readability** | |  |
|  | Simple Measure of Gobbledygook | .29 (-.01 - .59) |
|  | Flesch-Kincaid – online tool | .32 (.03 - .61) |
|  | Flesch-Kincaid – Microsoft Word | .32 (.03 - .61) |

95%CI: 95% confidence interval

Supplementary Table 2. Description of apps evaluated (N = 47).

| **Name of app** | **Platform available** | **Search term** | **Affiliations** | **Developers** | **HCPs involved** | **Languages** | **Purchasable content** | **Version** | **Last update** |
| --- | --- | --- | --- | --- | --- | --- | --- | --- | --- |
| ‎Aimee's Babies Newborn App | Apple | Infant activity | - | Comm. bus. developed by HCPs | OT | ENG | Free | 2.0 | 2013 |
| ‎Aimee's Babies Six Months | Apple | Infant activity | - | Comm. bus. developed by HCPs | OT | ENG | Free | 2.0 | 2014 |
| ‎Aimee's Babies Three Months | Apple | Infant activity | - | Comm. bus. developed by HCPs | OT | ENG | Free | 2.0 | 2014 |
| ‎Aimee's Babies Twelve Months | Apple | Infant activity | - | Comm. bus. developed by HCPs | OT | ENG | Free | 2.0 | 2014 |
| Annabel Karmel | Apple  Android | Infant feeding  Introducing solids | - | Comm. bus. consults with HCPs | Dr., DTN, LC, Nrs. | ENG | $5.99, $2.99 Fussy Eaters bundle | 2.4 (Apple)  1.16 (Android) | 2018 |
| Baby + - | Apple  Android | Infant feeding  Introducing solids  Infant activity | Comm. bus. | Unk. | - | DEU  DUT  ENG  FRA  ITA  POR  RUS  SPA | Free | 1.9.3 (Apple)  1.8.1 (Android) | 2018 |
| Baby Exercises & Activities | Apple  Android | Infant activity | Comm. bus. | Comm. bus. developed by HCPs | Sports therapist | ENG  SWE | Free with limited content,  $4.99 - $9.99 video access (Apple)  $5.99 - $8.99 (Android) | 2.0.4 | 2019 |
| Baby Food Chart | Android | Infant feeding  Introducing solids | - | Comm. bus. developed by HCPs | DTN | BOS  ENG  ITA | Free, $2.19 Premium | 1.2.9 | 2018 |
| Baby Led Kitchen | Android | Infant feeding  Introducing solids  Infant activity | - | Comm. bus. | - | ENG | $8.99 | 1.4.0 | 2018 |
| Baby Led Weaning - Guide & Recipes | Android | Infant feeding  Introducing solids | - | Unk. | - | DEU  ENG  FRA  ITA | Free, $4.99 Premium | 1.8 | 2018 |
| Baby solid food | Android | Infant feeding  Introducing solids | Unk. | Unk. | - | ENG | Free | 1.6 | 2017 |
| Baby weaning and recipes | Apple  Android | Infant feeding  Introducing solids | Comm. bus. | Comm. bus. | - | ENG | Free | 1.4.2 | 2018 |
| BabyGym | Apple  Android | Infant activity | - | Comm. bus. developed by HCPs | OT | ENG | Free with limited content,  $5.49 - $14.99 video access | 1.1 | 2018 |
| Baby-Led Weaning Recipes | Apple  Android | Infant feeding  Introducing solids  Infant activity | Unk. | Unk. | - | ENG | $8.49 | 1.8 | 2018 |
| BabySparks | Apple  Android | Infant activity | Comm. bus. | Comm. bus. consults with HCPs | Dr., OT, Psych., PT, SLP | ENG  SPA | Free with limited content,  $5.49 monthly - $49.99 lifetime Premium (Android)  $5.99 - $59.99 (Apple) | 2.2.29 (Apple)  2.2.26 (Android) | 2019 |
| ‎Best Breastfeeding Tips | Apple | Infant feeding | Unk. | Comm. bus. | - | ENG | Free, $0.99 Premium | 1.2 | 2014 |
| ‎Breast Baby - Feeding Pumping | Apple | Infant feeding | - | Unk. | - | ENG | $2.99 | 2.1.2 | 2018 |
| ‎Breast Start | Apple  Android | Infant feeding | - | Community health service | LC, Nrs. | ENG | Free | 1.8 | 2017 |
| Breastfeeding (Gateway Health) | Apple  Android | Infant feeding | NGO | Community health service | Health promotion | ENG | Free | 1.0 | 2018 |
| ‎Breastfeeding Central | Apple | Infant feeding | - | Comm. bus. developed by HCPs | LC | ENG | $5.99 | 1.01 | 2014 |
| ‎Breastfeeding Solutions | Apple  Android | Infant feeding | - | Comm. bus. developed by HCPs | LC | ENG | $7.99 | 1.3.2 | 2017 |
| Ella’s Kitchen First Foods | Apple  Android | Infant feeding  Introducing solids | - | Comm. bus. consults with HCPs | Nutritionist | ENG | Free with limited recipes, $6.49 | 2.1 | 2018 |
| ‎Feeding Friend | Apple | Infant feeding | - | Community health service | Midwife, Nrs. | ENG | Free | 1.4.0 | 2015 |
| Glow Baby | Apple  Android | Infant feeding  Introducing solids  Infant activity | Comm. bus. | Comm. bus. consults with HCPs | Dr., Nrs. | ENG | Free with breastfeeding articles content,  $7.99 monthly - $59.99 lifetime Premium (Apple)  $12.99 - $89.99 (Android) | 1.10.1 (Apple)  1.8.20 (Android) | 2018 |
| ‎Healthy Beginnings | Apple | Infant feeding | Govt.  Univ. | Community health service  Univ. | DTN, LC, Nrs. | ENG | Free | 1.1.3 | 2017 |
| ‎In Dads Care - Essential Baby Care for new Fathers | Apple  Android | Infant feeding | - | Comm. bus. | - | ENG | $2.99 | 1.3 | 2016 |
| Info for Nursing Mum | Apple  Android | Infant feeding | - | Community health service | Nrs. | CHI  ENG | Free | 1.0.4 | 2017 |
| Kinedu: Baby Development & Learning Activities | Apple  Android | Infant activity | Univ. | Comm. bus. consults with HCPs | Psych. | ENG  POR  SPA | Free with limited content,  $13.99 monthly - $229.99 lifetime Premium | 1.2.2. | 2018 |
| ‎latchME | Apple | Infant feeding | Comm. bus.  Govt.  NGO | Developed by HCPs | Dr., LC | ENG | Free | 3.1 | 2019 |
| Mamma Baby | Apple  Android | Infant feeding  Infant activity | - | Comm. bus. | - | ENG | Free, $4.99 (Android) or $5.99 (Apple) Premium | 2.0.2 (Apple)  0.1.2 (Android) | 2017 |
| ‎Move Baby Move | Apple | Infant activity | - | Comm. bus. developed by HCPs | PT | ENG | $5.99 | 1.1 | 2015 |
| My baby - Mother's vademecum | Apple | Infant feeding | - | Comm. bus. | - | ENG  POL | Free | 1.0.2 | 2018 |
| MyMedela Breastfeeding Companion | Apple  Android | Infant feeding  Infant activity | Comm. bus. | Comm. bus. consults with HCPs | LC, Nrs. | ENG | Free | 3.5.0 (Apple)  3.6.0 (Android) | 2018 |
| ‎NSH Baby Bump | Apple  Android | Infant feeding | - | Hospital | - | ENG | Free | 3.0.5 | 2018 |
| Ovia Parenting & Baby Development Tracker | Apple  Android | Infant feeding  Introducing solids  Infant activity | - | Comm. bus. developed by HCPs | Dr. | ENG | Free | 1.2.2 | 2018 |
| ‎Playfully Baby Development App | Apple  Android | Infant activity | - | Comm. bus. consults with HCPs | OT, SLP | ENG | Free trial, $10.99 3 months - $62.99 lifetime Premium | 1.3.1 (Apple)  1.1.1 (Android) | 2019 |
| PlayMama 0-1 year olds PRO | Android | Infant activity | - | Comm. bus. | - | ENG | $2.99 | 1.0-pro1 | 2015 |
| ‎Pregnancy & Baby Tracker | Apple  Android | Introducing solids  Infant activity | - | Comm. bus. consults with HCPs | Dr., Nrs. | CHI  DAN  DEU  ENG  FRA  JAP  POR  SPA | Free | 11.2.1 | 2019 |
| Rainbow - Journal & Activities | Android | Infant feeding  Infant activity | - | Unk. | - | ENG | Free, $2.89 ad-free | 2.5.9 | 2018 |
| Raising Children | Android | Infant feeding  Infant activity | Govt.  NGO | Comm. bus. consults with HCPs | Dr., health promotion, LC, midwife, Nrs., Psych. | ENG | Free | 1.3.2 | 2017 |
| ‎Red Nose Safe Sleeping | Apple  Android | Infant activity | Comm. bus. | NGO | - | ARA  CHI  ENG  HIN  VIE | Free | 3.2 | 2016 |
| Savvy Breastfeeding Guide | Apple  Android | Infant feeding | - | Comm. bus. developed by HCPs | LC | ENG | $2.43 | 1.1.8 | 2018 |
| ‎Super Baby | Apple | Infant activity | Unk. | Comm. bus. developed by HCPs | OT, PT | ENG | $4.49 | 1.5 | 2016 |
| Tummy Time Tracker - Improve Baby Coordination | Apple | Infant activity | Unk. | Comm. bus. | - | ENG | Free, $2.99 Premium | 1.1 | 2018 / 2017 (Pro) |
| WebMD Baby | Apple  Android | Infant feeding  Introducing solids  Infant activity | Comm. bus. | Comm. bus. developed by HCPs | Dr. | ENG | Free | 2.4 (Apple)  2.3.5 (Android) | 2018 |
| ‎WOT Baby | Apple | Infant feeding | - | Comm. bus. developed by HCPs | Nrs. | ENG | Free | 4.0 | 2017 |
| ‎WYNI - Breastfeeding Information | Apple  Android | Infant feeding | Govt.  Univ. | Community health service | Nrs. | ENG | Free | 1.3 | 2015 |

ARA: Arabic language

BOS: Bosnian language

CHI: Chinese language

Comm. bus.: commercial business

DAN: Danish language

DEU: German language

Dr.: doctor

DTN: dietitian

DUT: Dutch language

ENG: English language

FRA: French language

Govt.: government

HCPs: health care practitioners

HIN: Hindi language

ITA: Italian language

JAP: Japanese language

LC: lactation consultant

NGO: non-government organisation

Nrs.: nurse

OT: occupational therapy

POL: Polish language

POR: Portuguese language

Psych.: psychologist

PT: physiotherapist or physical therapist

RUS: Russian language

SLP: speech language therapist

SPA: Spanish language

SWE: Swedish language

Univ.: university

Unk.: unknown

VIE: Vietnamese language

- : nil or not available

Purchasable content listed in Australian dollars.


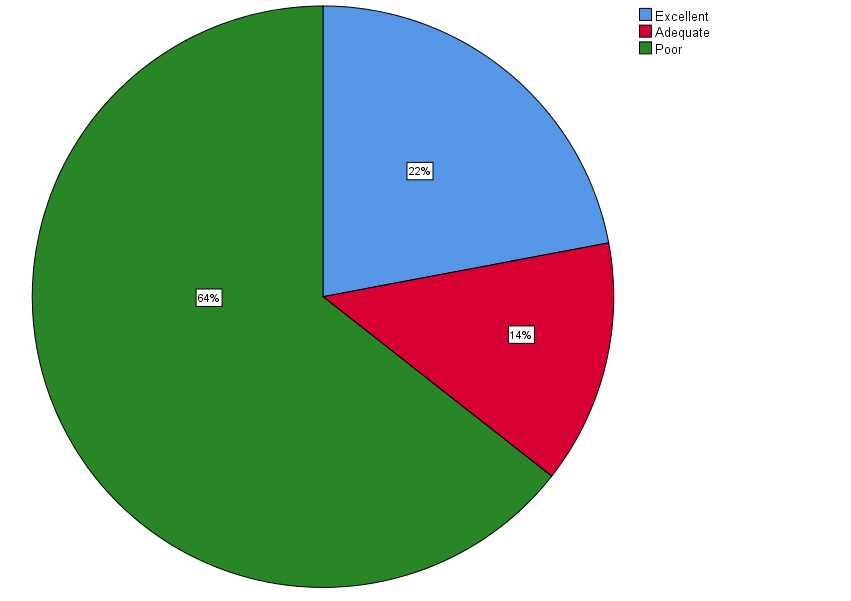


Supplementary Figure 1: Qualitative evaluation of coverage of information reported in all apps (N=59 evaluations for 47 apps).

Excellent: ≥90% coverage of information

Adequate: 75-89% coverage of information

Poor: ≤74% coverage of information


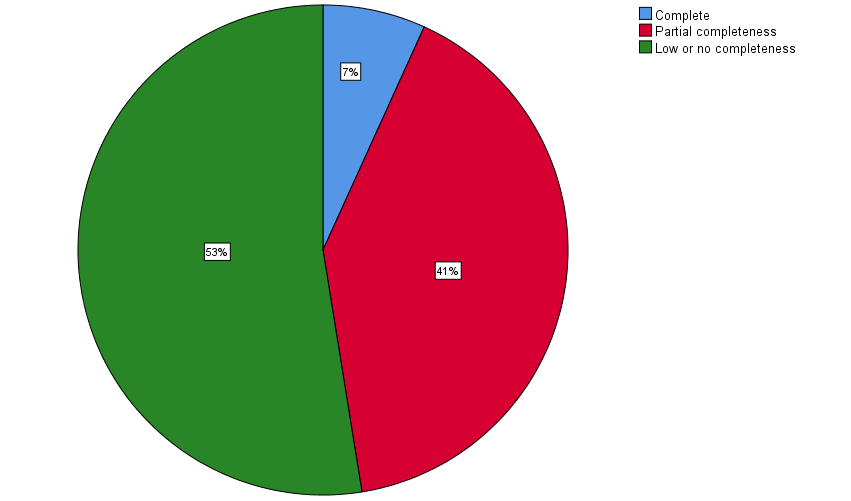


Supplementary Figure 2: Qualitative evaluation of depth of information reported in all apps (N=59 evaluations for 47 apps).

Complete: 100%

Partial completeness: 50-99%

Low or no completeness: ≤49%


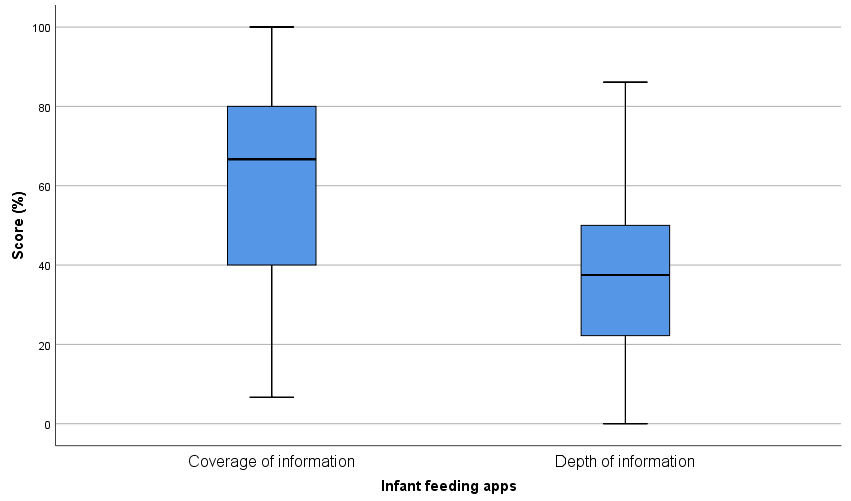
Supplementary Figure 3: Boxplot on coverage and depth of information reported in infant feeding apps.


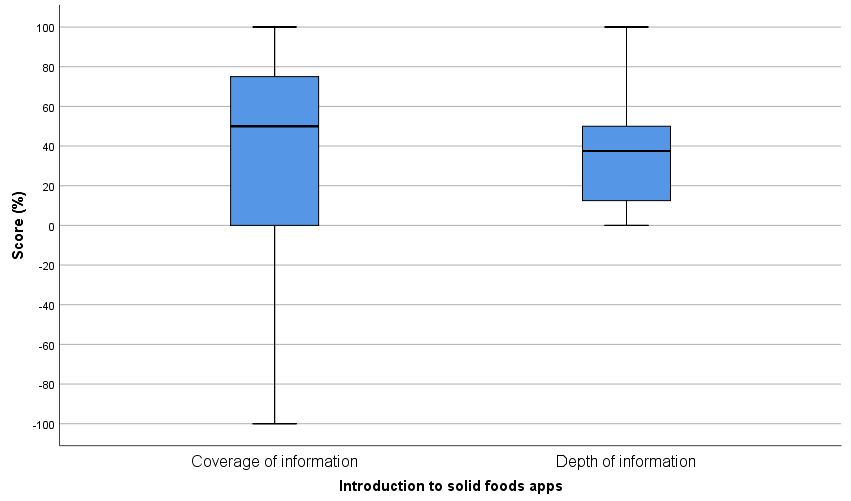


Supplementary Figure 4: Boxplot on coverage and depth of information reported in introduction to solids apps.


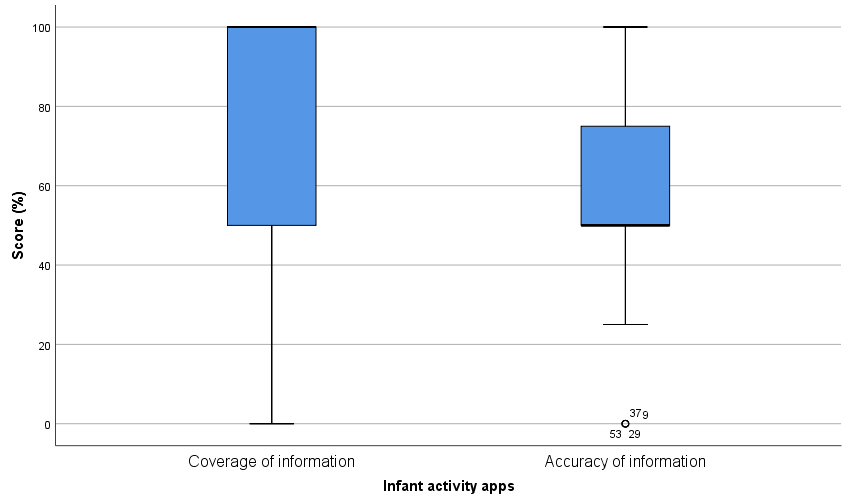


Supplementary Figure 5: Boxplot on coverage and depth of information reported in infant activity apps.


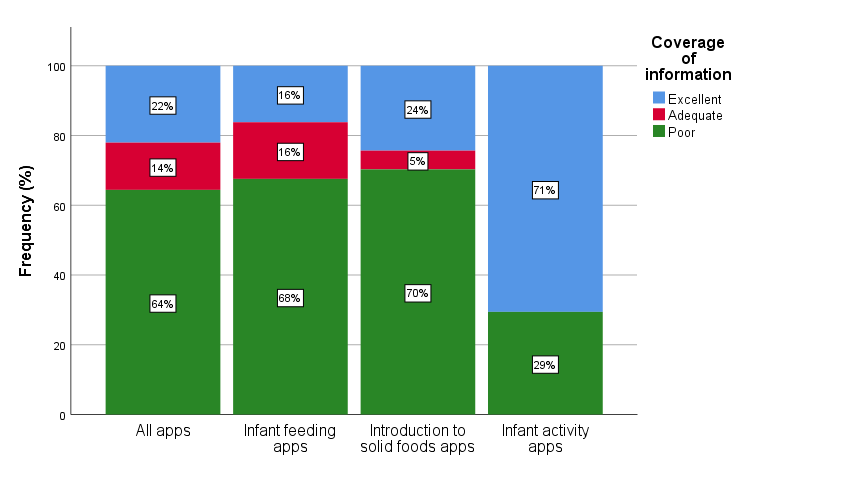


Supplementary Figure 6: Stacked bar graph on quality of coverage of information reported in all apps and across subtopics


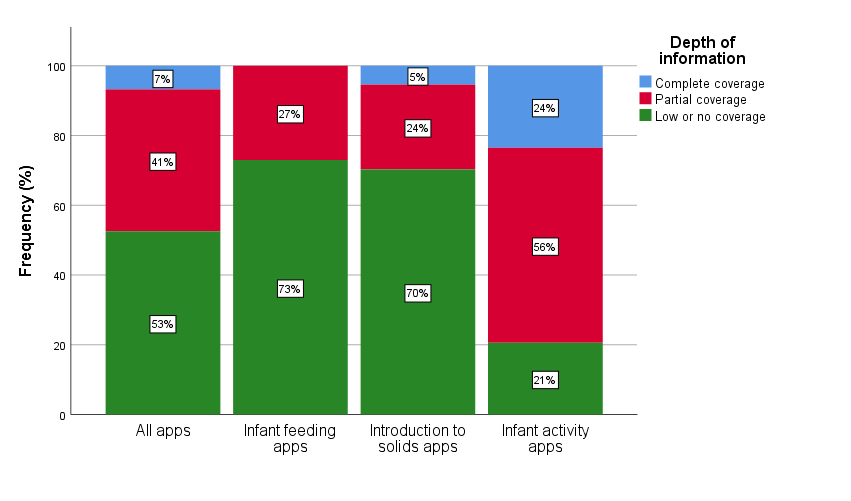


Supplementary Figure 7: Stacked bar graph on depth of information reported in all apps and across subtopics


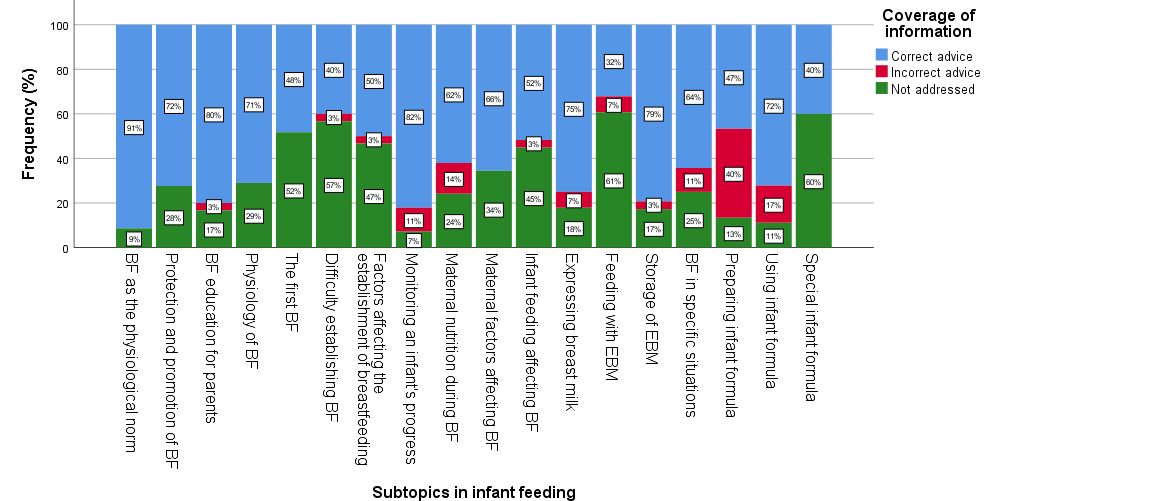


Supplementary Figure 8: Stacked bar graph on coverage of information reported across infant feeding subtopics.

BF: breastfeeding

EBM: expressed breast milk


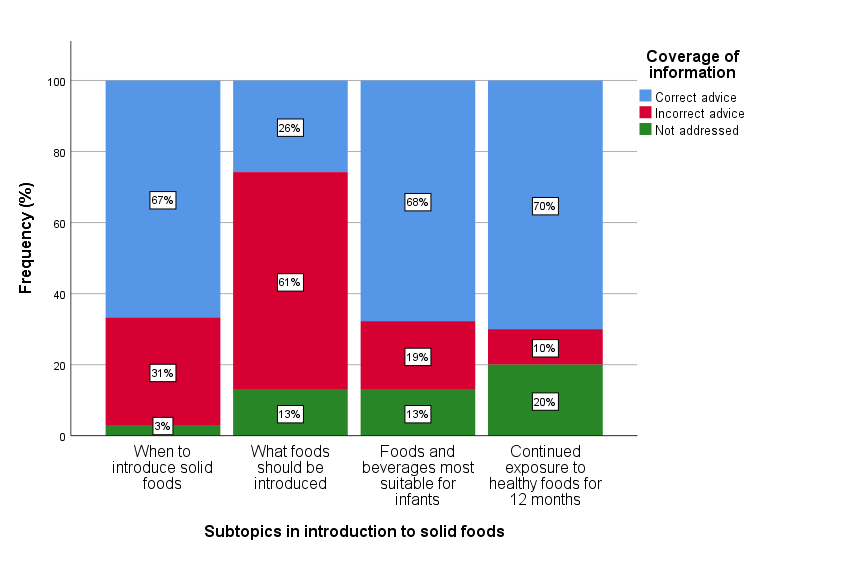


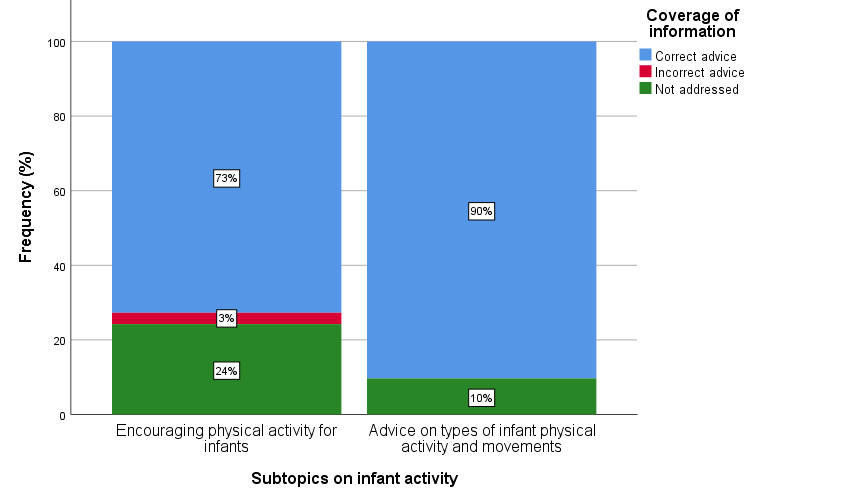
Supplementary Figure 9: Stacked bar graph on coverage of information reported across introduction to solids subtopics.

Supplementary Figure 10: Stacked bar graph on coverage of information reported across infant activity subtopics.


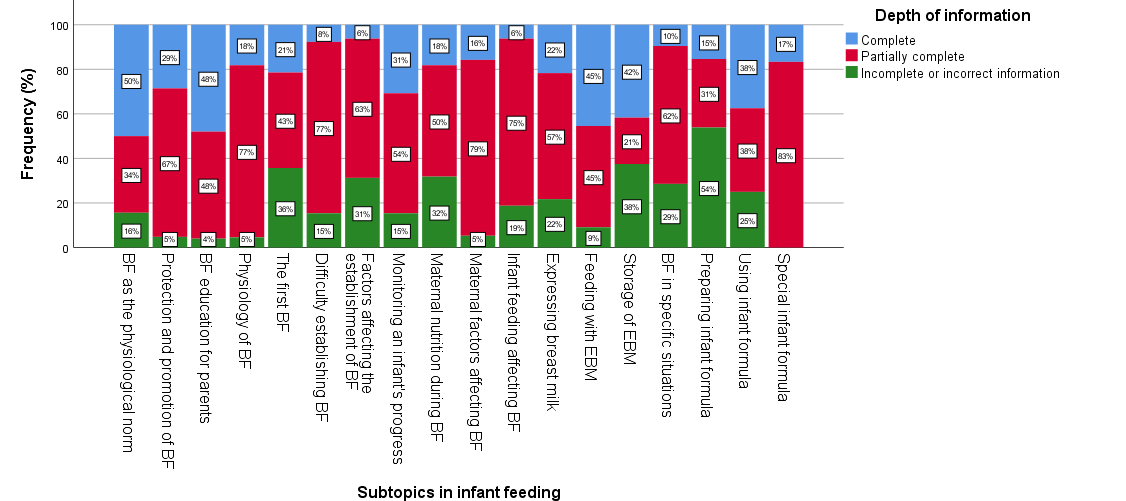


Supplementary Figure 11: Stacked bar graph on depth of information reported across infant feeding subtopics.

BF: breastfeeding

EBM: expressed breast milk


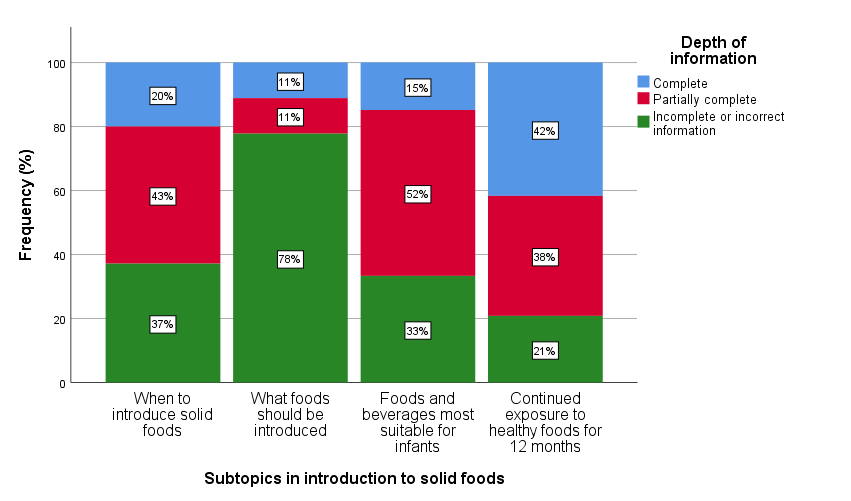
Supplementary Figure 12: Stacked bar graph on depth of information reported across introduction to solids subtopics.


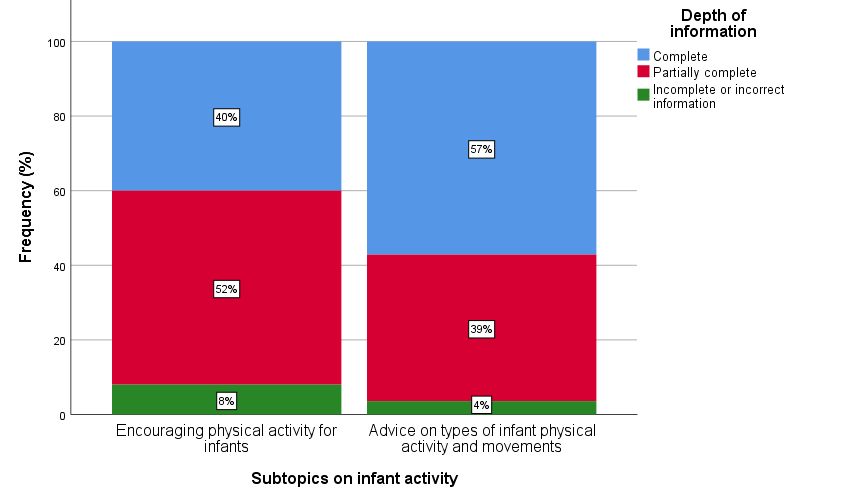


Supplementary Figure 13: Stacked bar graph on depth of information reported across infant activity subtopics.


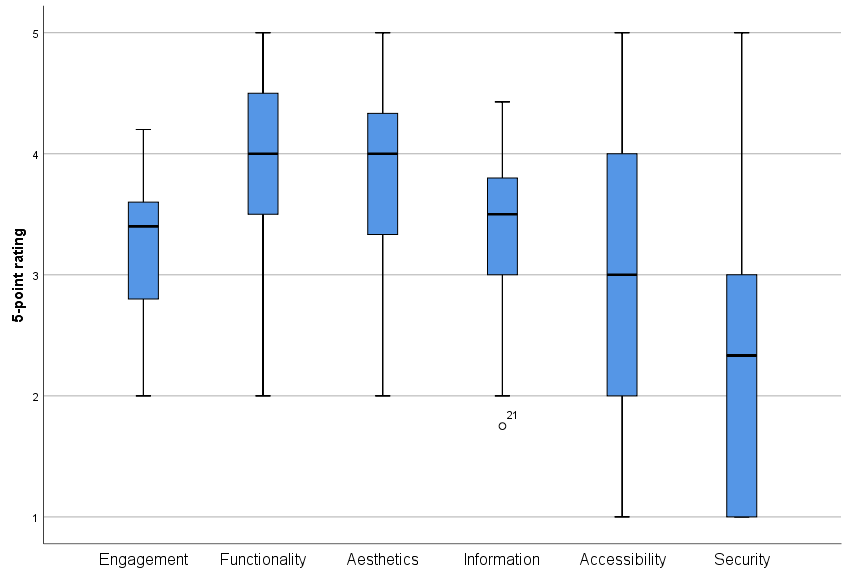


Supplementary Figure 14: 5-point rating scores for scales used to calculate the objective and modified MARS scores, with additional scales for accessibility and data security.


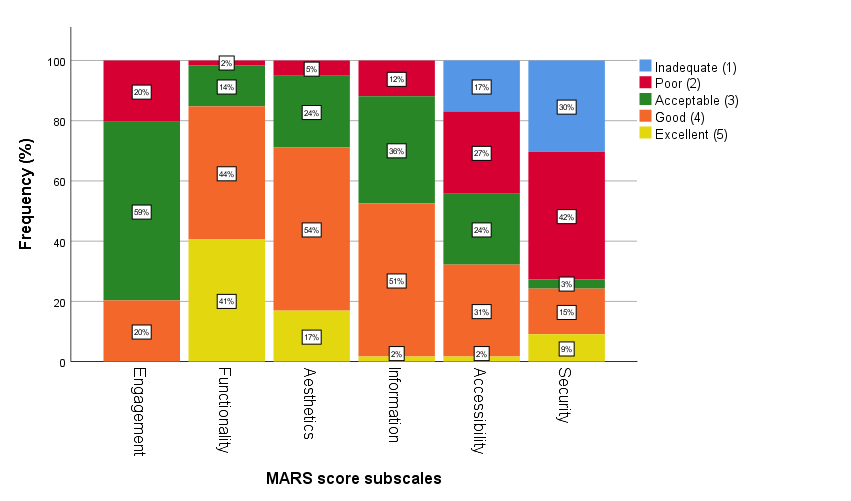


Supplementary Figure 15: Qualitative rating scores for subscales used to calculate the objective and modified MARS scores, with additional scales for accessibility and data security.

Supplementary Table 3. Correlation of 5-point app rating scores, from Google Play and App Store customer ratings and MARS evaluation.

|  | Subjective MARS score | Objective MARS score | Modified MARS score | Google Play rating | App Store rating |
| --- | --- | --- | --- | --- | --- |
| Subjective MARS score | - | - | - | - | - |
| MARS  score | .603^a^ | - | - | - | - |
| Modified MARS score | .546^a^ | .861^a^ | - | - | - |
| Google Play rating | -.293 | -.091 | .127 | - | - |
| App Store rating | -.026 | .254 | .056 | .388 | - |

^a^ Correlation is significant at the *P*<.001 level, two-tailed.

Supplementary Table 4. Individual Mobile App Rating Scores per smartphone app evaluation (N = 59 evaluations for 47 apps).

| **Name of app** | **Platform evaluated** | **Engagement scale** | **Functionality scale** | **Aesthetics subscale** | **Information scale** | **Accessibility scale** | **Security scale** | **Subjective MARS score** | **Objective MARS score** | **Modified MARS score** |
| --- | --- | --- | --- | --- | --- | --- | --- | --- | --- | --- |
| Aimee's Babies Newborn App | iOS | 3.20 | 5.00 | 3.67 | 4.25 | 2.33 | - | 2.25 | 4.03 | 3.69 |
| Aimee's Babies Six Months | iOS | 3.40 | 5.00 | 4.00 | 4.40 | 2.33 | 1.00 | 2.50 | 4.20 | 3.36 |
| Aimee's Babies Three Months | iOS | 3.00 | 4.75 | 4.67 | 4.40 | 3.00 | - | 4.25 | 4.20 | 3.96 |
| Aimee's Babies Twelve Months | iOS | 2.80 | 5.00 | 4.67 | 4.00 | 2.33 | - | 2.50 | 4.12 | 3.76 |
| Annabel Karmel | Android | 3.60 | 4.50 | 4.67 | 3.40 | 3.00 | - | 2.50 | 4.04 | 3.83 |
| Annabel Karmel | iOS | 3.20 | 4.50 | 4.67 | 3.60 | 2.00 | - | 2.75 | 3.99 | 3.59 |
| Baby +- | iOS | 3.60 | 4.75 | 4.67 | 3.60 | 4.00 | 2.33 | 3.00 | 4.15 | 3.82 |
| Baby +- | Android | 4.20 | 4.75 | 4.00 | 3.60 | 3.00 | 2.33 | 2.00 | 4.14 | 3.65 |
| Baby Exercises & Activities | iOS | 2.80 | 5.00 | 4.00 | 3.00 | 3.67 | - | 4.00 | 3.70 | 3.69 |
| Baby Exercises & Activities | Android | 2.60 | 4.50 | 4.00 | 3.40 | 3.67 | - | 1.50 | 3.63 | 3.63 |
| Baby Food Chart | Android | 2.60 | 3.25 | 4.00 | 2.50 | 4.00 | 1.00 | 2.50 | 3.09 | 2.89 |
| Baby Led Kitchen | Android | 2.40 | 4.25 | 4.00 | 2.40 | 2.33 | - | 2.25 | 3.26 | 3.08 |
| Baby Led Weaning - Guide & Recipes | Android | 2.40 | 4.75 | 3.67 | 3.40 | 2.33 | - | 2.25 | 3.55 | 3.31 |
| Baby solid food | Android | 2.00 | 4.25 | 2.67 | 2.50 | 1.00 | - | 1.00 | 2.85 | 2.48 |
| Baby weaning and recipes | iOS | 3.20 | 5.00 | 5.00 | 3.80 | 3.67 | 5.00 | 3.75 | 4.25 | 4.28 |
| Baby weaning and recipes | Android | 2.80 | 4.00 | 4.33 | 3.40 | 3.67 | 3.00 | 3.75 | 3.63 | 3.53 |
| BabyGym | Android | 3.20 | 4.50 | 3.67 | 4.00 | 1.00 | - | 4.00 | 3.84 | 3.27 |
| Baby-Led Weaning Recipes | Android | 2.40 | 4.00 | 4.00 | 2.25 | 3.00 | - | 1.50 | 3.16 | 3.13 |
| BabySparks | Android | 2.80 | 3.75 | 4.67 | 3.67 | 5.00 | 1.00 | 3.50 | 3.72 | 3.48 |
| BabySparks | iOS | 2.80 | 4.00 | 3.33 | 4.20 | 3.67 | - | 3.50 | 3.58 | 3.60 |
| Best Breastfeeding Tips | iOS | 2.20 | 4.00 | 3.00 | 1.75 | 1.00 | 1.00 | 1.25 | 2.74 | 2.16 |
| Breast Baby - Feeding Pumping | iOS | 3.20 | 4.00 | 2.67 | 3.60 | 3.00 | 1.00 | 3.00 | 3.37 | 2.91 |
| Breast Start | iOS | 2.60 | 3.50 | 2.67 | 3.60 | 1.00 | - | 3.75 | 3.09 | 2.67 |
| Breastfeeding | iOS | 2.40 | 4.75 | 4.00 | 3.75 | 3.00 | - | 2.00 | 3.73 | 3.58 |
| Breastfeeding | Android | 2.40 | 4.75 | 4.33 | 3.25 | 1.00 | - | 2.25 | 3.68 | 3.15 |
| Breastfeeding Central | iOS | 1.80 | 4.00 | 2.00 | 3.00 | 3.67 | - | 1.50 | 2.70 | 2.89 |
| Breastfeeding Solutions | iOS | 1.80 | 3.25 | 2.67 | 4.00 | 3.00 | - | 2.00 | 2.93 | 2.94 |
| Ella's Kitchen First Foods | Android | 2.80 | 4.00 | 4.00 | 3.80 | 2.33 | - | 3.50 | 3.65 | 3.39 |
| Feeding Friend | iOS | 2.80 | 4.00 | 3.33 | 3.00 | 4.00 | 1.00 | 1.25 | 3.28 | 3.02 |
| Glow Baby | Android | 3.20 | 4.75 | 4.00 | 3.60 | 4.00 | 2.33 | 2.50 | 3.89 | 3.65 |
| Glow Baby | iOS | 3.80 | 4.50 | 4.00 | 3.00 | 4.00 | 2.33 | 1.75 | 3.83 | 3.61 |
| Healthy Beginnings | iOS | 3.60 | 3.75 | 4.33 | 3.40 | 2.33 | 5.00 | 2.50 | 3.77 | 3.74 |
| In Dads Care - Essential Baby Care for new Fathers | iOS | 2.40 | 4.25 | 3.67 | 3.00 | 1.00 | - | 1.50 | 3.33 | 2.86 |
| Info for Nursing Mum | Android | 3.00 | 3.75 | 3.33 | 4.40 | 3.67 | 5.00 | 1.75 | 3.62 | 3.86 |
| Kinedu: Baby Development and Learning Activities | Android | 3.80 | 2.75 | 4.33 | 3.50 | 4.00 | 2.33 | 3.00 | 3.60 | 3.45 |
| latchME | Android | 2.00 | 3.00 | 2.00 | 2.40 | 2.00 | 1.00 | 1.00 | 2.35 | 2.07 |
| Mamma Baby | iOS | 2.60 | 3.00 | 3.00 | 2.00 | 3.00 | 2.33 | 1.25 | 2.65 | 2.66 |
| Mamma Baby | Android | 2.60 | 2.00 | 3.33 | 2.20 | 3.00 | 2.33 | 1.00 | 2.53 | 2.58 |
| Move Baby Move | iOS | 2.60 | 2.50 | 3.67 | 3.33 | 1.00 | - | 2.75 | 3.03 | 2.62 |
| My baby - Mother's vademecum | iOS | 3.40 | 4.50 | 3.67 | 2.83 | 4.00 | 1.00 | 1.25 | 3.60 | 3.23 |
| MyMedela Breastfeeding Companion | Android | 3.20 | 2.75 | 3.67 | 3.00 | 3.00 | 2.33 | 3.00 | 3.15 | 2.99 |
| NSH Baby Bump | iOS | 3.60 | 4.50 | 4.67 | 4.00 | 1.00 | 1.00 | 4.00 | 4.19 | 3.13 |
| Ovia Parenting & Baby Development Tracker | Android | 3.40 | 4.50 | 4.33 | 4.00 | 4.00 | 2.33 | 3.25 | 4.06 | 3.76 |
| Playfully Baby | iOS | 3.40 | 4.75 | 5.00 | 3.67 | 2.00 | 2.33 | 2.75 | 4.20 | 3.53 |
| Playfully Baby | Android | 3.20 | 4.25 | 4.33 | 3.60 | 3.00 | 2.33 | 3.25 | 3.85 | 3.45 |
| PlayMama 0-1 year olds PRO | Android | 2.60 | 4.00 | 3.33 | 2.40 | 1.00 | 2.33 | 2.00 | 3.08 | 2.61 |
| Pregnancy and Baby Tracker | Android | 3.60 | 4.25 | 3.67 | 3.20 | 2.00 | 3.67 | 2.50 | 3.68 | 3.40 |
| Pregnancy and Baby Tracker | iOS | 3.80 | 3.50 | 3.67 | 3.40 | 2.00 | 3.67 | 3.75 | 3.59 | 3.34 |
| Rainbow - Journal & Activities | Android | 3.40 | 4.00 | 4.00 | 2.80 | 4.00 | 3.67 | 2.25 | 3.55 | 3.64 |
| Raising Children | Android | 3.20 | 4.25 | 4.00 | 3.60 | 2.33 | - | 3.75 | 3.76 | 3.48 |
| Red Nose Safe Sleeping | iOS | 2.80 | 4.75 | 4.33 | 4.80 | 3.67 | - | 4.00 | 4.17 | 4.07 |
| Savvy Breastfeeding Guide | iOS | 2.00 | 3.50 | 2.00 | 3.00 | 1.00 | - | 1.00 | 2.63 | 2.30 |
| Super Baby - Video center for infant development to reach gross motor skills and baby milestones. | iOS | 3.40 | 4.00 | 4.00 | 3.75 | 4.00 | - | 3.75 | 3.79 | 3.83 |
| Tummy Time Tracker | iOS | 3.40 | 3.50 | 2.67 | 3.40 | 3.00 | 2.33 | 2.00 | 3.24 | 3.05 |
| Tummy Time Tracker Pro | iOS | 3.00 | 4.00 | 4.00 | 3.40 | 2.00 | 2.33 | 2.75 | 3.60 | 3.12 |
| WebMD Baby | iOS | 3.60 | 3.75 | 3.00 | 4.20 | 3.00 | 3.67 | 3.50 | 3.64 | 3.54 |
| WebMD Baby | Android | 3.40 | 3.25 | 3.33 | 3.80 | 3.00 | 3.67 | 3.25 | 3.45 | 3.41 |
| WOT Baby | iOS | 3.80 | 4.75 | 4.00 | 3.50 | 2.33 | - | 2.25 | 4.01 | 3.68 |
| WYNI - Breastfeeding Information | iOS | 4.20 | 5.00 | 4.67 | 4.43 | 2.00 | 1.00 | 3.75 | 4.57 | 3.55 |

Supplementary Table 5. Suitability of health information using the SAM tool (N = 59 evaluations for 47 apps).

|  | | **SAM scores, n evaluations (%)** | | | |
| --- | --- | --- | --- | --- | --- |
|  | | **Not suitable** | **Adequate** | **Superior** | **Not applicable** |
|  | |  |  |  |  |
| **Content** | | | | | |
|  | Purpose is evident | 1 (2) | 26 (44) | 30 (51) | 2 (3) |
|  | Content about behaviours | 2 (3) | 34 (58) | 22 (37) | 1 (2) |
|  | Limited to essential information | 0 | 20 (34) | 39 (66) | 0 |
|  | Summary and review | 6 (10) | 6 (10) | 3 (5) | 44 (75) |
| **Literacy demand** | | | | | |
|  | Reading grade level | 22 (37) | 27 (46) | 10 (17) | 0 |
|  | Writing style with active voice | 2 (3) | 18 (31) | 38 (64) | 1 (2) |
|  | Vocabulary uses common words | 1 (2) | 31 (53) | 26 (44) | 1 (2) |
|  | Context given first | 7 (12) | 28 (48) | 17 (29) | 7 (12) |
|  | Headers or topic captions | 4 (7) | 23 (39) | 20 (34) | 12 (20) |
| **Graphics** | | | | | |
|  | Purposeful cover graphic | 2 (3) | 31 (53) | 13 (22) | 13 (22) |
|  | Appropriate type of illustrations | 2 (3) | 7 (12) | 8 (14) | 42 (71) |
|  | Relevance of illustrations | 3 (5) | 6 (10) | 8 (14) | 42 (71) |
|  | Lists, tables, graphs and charts explained | 0 | 1 (2) | 0 | 58 (98) |
|  | Captions used for graphics | 0 | 3 (5) | 1 (2) | 55 (93) |
| **Layout and typography** | | | | | |
|  | Layout factors | 4 (7) | 32 (54) | 20 (34) | 3 (5) |
|  | Typography | 3 (5) | 35 (59) | 19 (32) | 2 (3) |
|  | Subheadings used | 5 (9) | 15 (25) | 25 (42) | 14 (24) |
| **Learning, stimulation and motivation** | | | | | |
|  | Interaction with readers used | 13 (22) | 33 (56) | 6 (10) | 7 (12) |
|  | Modelled and specific behaviours | 4 (7) | 17 (29) | 35 (59) | 3 (5) |
|  | Self-efficacious tasks and behaviours | 2 (3) | 21 (36) | 32 (54) | 4 (7) |
| **Cultural appropriateness** | | | | | |
|  | Cultural match | 0 | 56 (95) | 3 (5) | 0 |
|  | Cultural image and examples | 0 | 0 | 3 (5) | 56 (95) |

Supplementary Table 6. Individual Suitability Assessment of Materials scores per smartphone app evaluation (N = 59 evaluations for 47 apps).

| **Name of app** | **Platform evaluated** | **Content  (max score /8)** | **Literacy demand  (max score /10)** | **Graphics  (max score /10)** | **Layout and terminology  (max score /6)** | **Learning, stimulation and motivation  (max score /6)** | **Cultural appropriateness (max score /4)** | **SAM score** | **Possible points** | **SAM rating** |
| --- | --- | --- | --- | --- | --- | --- | --- | --- | --- | --- |
| Aimee's Babies Newborn App | iOS | 4 | 8 | - | 1 | 3 | 3 | 19 | 26 | Superior |
| Aimee's Babies Six Months | iOS | 6 | 6 | - | 1 | 5 | 1 | 19 | 26 | Superior |
| Aimee's Babies Three Months | iOS | 6 | 10 | - | 3 | 5 | 3 | 27 | 30 | Superior |
| Aimee's Babies Twelve Months | iOS | 4 | 8 | - | 2 | 3 | 3 | 20 | 28 | Superior |
| Annabel Karmel | iOS | 4 | 5 | 1 | 3 | 4 | 2 | 19 | 34 | Adequate |
| Annabel Karmel | Android | 4 | 6 | 1 | 3 | 4 | 1 | 19 | 32 | Adequate |
| Baby +- | Android | 5 | 6 | 2 | 5 | 4 | 1 | 23 | 32 | Superior |
| Baby +- | iOS | 6 | 8 | 2 | 2 | 4 | 1 | 23 | 34 | Adequate |
| Baby Exercises & Activities | Android | 5 | 3 | 1 | 2 | 3 | 1 | 15 | 26 | Adequate |
| Baby Exercises & Activities | iOS | 4 | 5 | 1 | - | 2 | 1 | 13 | 18 | Superior |
| Baby Food Chart | Android | 3 | 1 | 2 | 1 | 1 | 3 | 11 | 34 | Not suitable |
| Baby Led Kitchen | Android | 4 | 5 | 2 | 6 | 3 | 1 | 21 | 30 | Superior |
| Baby Led Weaning - Guide & Recipes | Android | 3 | 4 | 1 | 3 | 4 | 1 | 16 | 32 | Adequate |
| Baby solid food | Android | 5 | 4 | - | 2 | 5 | 1 | 17 | 30 | Adequate |
| Baby weaning and recipes | Android | 4 | 5 | 1 | 6 | 5 | 1 | 22 | 32 | Adequate |
| Baby weaning and recipes | iOS | 5 | 4 | 1 | 6 | 5 | 2 | 23 | 34 | Adequate |
| Baby-Led Weaning Recipes | Android | 3 | 5 | - | 3 | 3 | 2 | 16 | 32 | Adequate |
| BabyGym | Android | 4 | 3 | 1 | 3 | 3 | 1 | 15 | 32 | Adequate |
| BabySparks | Android | 4 | 7 | 0 | 6 | 2 | 1 | 22 | 36 | Adequate |
| BabySparks | iOS | 4 | 6 | 4 | 3 | 4 | 1 | 20 | 38 | Adequate |
| Best Breastfeeding Tips | iOS | 5 | 7 | 1 | 3 | 3 | 1 | 20 | 34 | Adequate |
| Breast Baby - Feeding Pumping | iOS | 6 | 7 | - | 4 | 3 | 1 | 21 | 30 | Superior |
| Breast Start | iOS | 4 | 8 | - | 3 | 5 | 1 | 21 | 30 | Superior |
| Breastfeeding | Android | 7 | 7 | 1 | 5 | 4 | 1 | 25 | 34 | Superior |
| Breastfeeding | iOS | 7 | 8 | 2 | 4 | 5 | 1 | 27 | 34 | Superior |
| Breastfeeding Central | iOS | 4 | 5 | 3 | 2 | 2 | 1 | 17 | 34 | Adequate |
| Breastfeeding Solutions | iOS | 4 | 5 | 3 | 3 | - | 1 | 16 | 34 | Adequate |
| Ella's Kitchen First Foods | Android | 4 | 6 | - | 4 | 5 | 1 | 20 | 30 | Adequate |
| Feeding Friend | iOS | 4 | 7 | - | 5 | 2 | 1 | 19 | 30 | Adequate |
| Glow Baby | Android | 5 | 6 | 1 | 3 | 5 | 1 | 21 | 32 | Adequate |
| Glow Baby | iOS | 3 | 2 | 1 |  | 1 | 1 | 8 | 12 | Adequate |
| Healthy Beginnings | iOS | 3 | 3 | 1 | 3 | 3 | 2 | 15 | 30 | Adequate |
| In Dads Care - Essential Baby Care for new Fathers | iOS | 5 | 5 | 3 | 3 | - | 1 | 17 | 32 | Adequate |
| Info for Nursing Mum | Android | 8 | 7 | 1 | 6 | 5 | 4 | 31 | 36 | Superior |
| Kinedu: Baby Development and Learning Activities | Android | 5 | 7 | 2 | 3 | 5 | 1 | 23 | 32 | Superior |
| latchME | Android | 3 | 4 | 0 | 1 | 2 | 1 | 11 | 30 | Not suitable |
| Mamma Baby | Android | 4 | 5 | - | 5 | 3 | 1 | 18 | 28 | Adequate |
| Mamma Baby | iOS | 4 | 6 | 1 | 2 | 3 | 1 | 17 | 34 | Adequate |
| Move Baby Move | iOS | 4 | 5 | 6 | 3 | 3 | 1 | 22 | 38 | Adequate |
| My baby - Mother's vademecum | iOS | 5 | 5 | 5 | 5 | 1 | 1 | 22 | 36 | Adequate |
| MyMedela Breastfeeding Companion | Android | 6 | 6 | 5 | 5 | 6 | 1 | 29 | 36 | Superior |
| NSH Baby Bump | iOS | 5 | 9 | 1 | 6 | 6 | 1 | 28 | 36 | Superior |
| Ovia Parenting & Baby Development Tracker | Android | 6 | 7 | 2 | 6 | 4 | 1 | 26 | 32 | Superior |
| Playfully Baby | Android | 6 | 5 | 2 | 3 | 6 | 1 | 23 | 26 | Superior |
| Playfully Baby | iOS | 3 | 5 | 3 | 2 | 3 | 1 | 17 | 34 | Adequate |
| PlayMama 0-1 year olds PRO | Android | 5 | 6 | 6 | 6 | 4 | 1 | 28 | 32 | Superior |
| Pregnancy and Baby Tracker | Android | 3 | 6 | 1 | 6 | 4 | 1 | 22 | 32 | Adequate |
| Pregnancy and Baby Tracker | iOS | 3 | 9 | 1 | 4 | 4 | 1 | 21 | 32 | Adequate |
| Rainbow - Journal & Activities | Android | 6 | 8 | 1 | 3 | 5 | 1 | 24 | 30 | Superior |
| Raising Children | Android | 6 | 5 | 2 | 5 | 5 | 4 | 27 | 32 | Superior |
| Red Nose Safe Sleeping | iOS | 6 | 10 | 7 | 6 | 6 | 2 | 37 | 40 | Superior |
| Savvy Breastfeeding Guide | iOS | 4 | 4 | 5 | 3 | 2 | 1 | 19 | 40 | Adequate |
| Super Baby - Video center for infant development to reach gross motor skills and baby milestones. | iOS | 6 | 4 | 2 | 4 | 4 | 1 | 21 | 24 | Superior |
| Tummy Time Tracker | iOS | 6 | 5 | 4 | 2 | 5 | 1 | 23 | 32 | Superior |
| Tummy Time Tracker Pro | iOS | 4 | 8 | 3 | 2 | 3 | 1 | 21 | 36 | Adequate |
| WebMD Baby | Android | 4 | 7 | - | 5 | 4 | 1 | 21 | 30 | Superior |
| WebMD Baby | iOS | 3 | 8 | 1 | 4 | 4 | 1 | 21 | 32 | Adequate |
| WOT Baby | iOS | 5 | 4 | 6 | 4 | 4 | 1 | 24 | 36 | Adequate |
| WYNI - Breastfeeding Information | iOS | 6 | 9 | 5 | 6 | 6 | 1 | 33 | 38 | Superior |

Maximum score refers to total score in each section for applicable factors. Factors scored as ‘not applicable’ are not scored when calculating score for each section, or for the overall SAM rating.

The SAM rating is calculated as total of SAM score (sum of content, literacy demand, graphics, layout and terminology, learning, stimulation and motivation, and cultural appropriateness scores) divided by possible points, with superior as 70-100%, adequate as 40-69%, or not suitable as 0-39%.

Supplementary Table 7. Correlation of readability scores of infant feeding and activity apps.

|  | F-K score – online | F-K score – Word | SMOG score |
| --- | --- | --- | --- |
|  |  |  |  |
| F-K score – online | - | - | - |
| F-K score – Word | .880^a^ | - | - |
| SMOG score | .862^a^ | .810^a^ | - |

^a^ Correlation is significant at the *P*<.001 level, two-tailed.

F-K: Flesch-Kincaid

SMOG: Simple Measure of Gobbledygook
